# Supplementary material for: Towards monitoring of global health research: an exploratory analysis of transparency and stakeholder engagement
Source: BMJ Open. 2025 Dec 12;15(12):e102010. doi: 10.1136/bmjopen-2025-102010 (PMC12706105; doi:10.1136/bmjopen-2025-102010)
Supplement: Supplementary data [file bmjopen-15-12-s001.pdf]

Supplementary file

Supplement S1: Search strategies to identify global health studies

1**. Clinical trial registry with a disease-based approach**

On 13th April 2024, using the ClinicalTrials.gov web front (https://clinicaltrials.gov/), we searched studies by Condition/disease (MeSH terms) in the following way:

Condition	N
Postpartum Depression (also searched for **Depressive Disorders**, **Puerperal**, and **Post Natal Depression**. See Search Details)	450
Maternal Sepsis (also searched for **Depressive Disorders**, **Puerperal**, and **Post Natal Depression**. See Search Details)	1403
Maternal Anemia (also searched for **Anemia of pregnancy**, **Pregnant**, **Anemia during pregnancy** and more. See Search Details)	437
Tuberculosis (See Search Details)	1391

2. **Global Health Journal**

On 13th April 2024, using the Cochrane Highly Sensitive Search Strategy (Box 3b strategy), we identified randomized trials in PubMed and cross-referenced these results with articles from 20 global health journals.

Box 3. b Cochrane Highly Sensitive Search Strategy for identifying randomized trials in MEDLINE: sensitivity- and precision-maximizing version (2008 revision); PubMed format

#1 randomized controlled trial [pt]

#2 controlled clinical trial [pt]

#3 randomized [tiab]

#4 placebo [tiab]

#5 clinical trials as topic [mesh:noexp]

#6 randomly [tiab]

#7 trial [ti]

#8 #1 OR #2 OR #3 OR #4 OR #5 OR #6 OR #7

#9 animals [mh] NOT humans [mh]

#10 #8 NOT #9

PubMed search syntax:
[pt] denotes a Publication Type term;
[tiab] denotes a word in the title or abstract;
[sh] denotes a subheading;
[mh] denotes a Medical Subject Heading (MeSH) term ‘exploded’;
[mesh:noexp] denotes a Medical Subject Heading (MeSH) term not ‘exploded’;
[ti] denotes a word in the title.

Supplementary file

PubMed terms used to extract articles

((((("The Lancet. Global health"[Journal]) OR ("Journal of global health"[Journal])) OR ("Globalization and health"[Journal])) OR ("Annals of global health"[Journal])) OR ("pathogens and global health"[Journal])) OR ("Global public health"[Journal])) OR ("Global health action"[Journal])) OR ("Global health promotion"[Journal])) OR ("BMJ global health"[Journal])) OR ("Global pediatric health"[Journal])) OR ("Global health, epidemiology and genomics"[Journal])) OR ("Global health research and policy"[Journal])) OR ("The Central African journal of medicine"[Journal])) OR ("Clinical epidemiology and global health"[Journal])) OR ("Global journal of health science"[Journal])) OR ("Global mental health (Cambridge, England)"[Journal])) OR ("international journal of travel medicine and global health"[Journal])) OR ("plos global public health"[Journal])) OR ("Global journal of health science"[Journal])) OR ("journal of global health perspectives"[Journal])) OR ("Global health journal (Amsterdam, Netherlands)"[Journal])) and (((randomized controlled trial[Publication Type]) OR (controlled clinical trial[Publication Type]) OR (randomized[Title/Abstract]) OR (placebo[Title/Abstract]) OR (clinical trials as topic[mesh:noexp]) OR (randomly[Title/Abstract]) OR (randomly[Title/Abstract]) OR (trial[Title])) NOT (animals [mh] NOT humans [mh]))) NOT review [pt]) AND ((english[Filter]) AND (2011:2024[pdat]))) Filters: English, from 2011 – 2024

Note: We additionally excluded articles (n = 9) from “The Central African journal of medicine” journal, which was not explicitly a global health journal. Please see “13-4-2024-global-health-article” for details.

Supplementary file

Supplement S1 Global health journal analysis flowchart

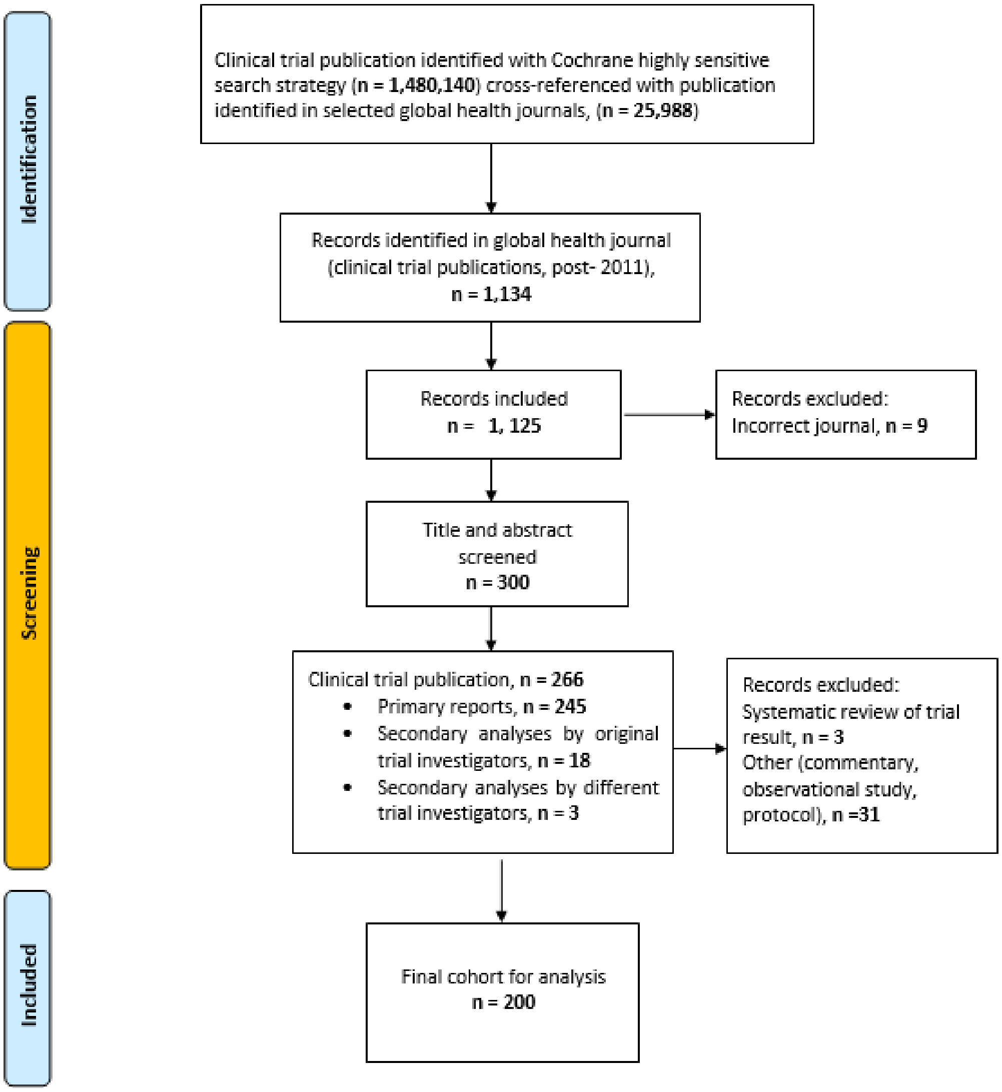

Supplementary file

3. Global health funder website

On 13th April 2024, using the NIH RePORTER tool's 'Advanced Project Search' (https://reporter.nih.gov/advanced-search) and selecting the Funding tab, with Agency/Institute/Center set to "John E. Fogarty International Center for Advanced Study in the Health Sciences (FIC)," the search results included:

- 73 clinical trials,
- 9,515 publications,
- Supported by 331 core projects.

Supplement S2: Search manual for clinical trial result publication

Our goal is to identify result publications for clinical trials using the criteria mentioned in this manual. We will strive to find the earliest result publication that matches the trial in a reasonably unambiguous way and reports its results.

Identification steps

Identification of publications linked to each clinical trial will be done in a 3-step process. Go through steps 1-2 for all trials. Continue with step 3 only if no eligible results publication has been found during steps 1-2.

Check the ClinicalTrials.gov website under the tab 'Publications' to identify results publications linked in the registration. At ClinicalTrials.gov, these are either actively submitted by the sponsor/investigator or automatically indexed. Links may lead to reviews or other background literature, which will not be counted as results publications.

2) Google search using the main NCT ID. The first page (or a minimum of 10 first hits) will be screened.

3) Google search using two combinations of terms from the registry, e.g., title, principal investigator name, intervention/treatment, disease/symptoms, or other. Use combinations that you believe are specific enough to yield a valid match. The first page will be screened for each combination.

At each step, search for eligible results publication, except those with the same URL or same DOI as one previously extracted for the same trial. Among unique eligible result publications found at each step, enter only one with the earliest publication date.

Earliest Publication date**?:** The earliest date an article was published. Make sure you are checking to see if there is an "Epub" or "Online First" date (sometimes you need to check both PubMed and the Journal website) because, for a lot of journals, there will be both the date it was first published online and then a subsequent date it was published in print (for journals that still do print issues!) We want the earliest it shows up in any format.

Supplementary file

For all steps, if a publication is found it will be verified that it is indeed a results publication for the study and not only a mention of the NCT ID in a different context (e.g. review listingto th ongoing or completed trials). This verification is performed based on the title and abstract of the publication and if needed by referring to the full text. If the publication is not a results publication, the publication search will continue. If no hit occurs, we will proceed to the next step. Only if all three searches stay without results, the study will be characterized as “no publication found”.

Extractions from publications

In case we identify a publication, we will extract manually the following identifiers: PubMed ID, DOI, URL and the earliest publication date. If only month and year are given, we will impute the day as the first day of the month.

Interrater reliability

Two reviewers (NH and SSY) will initially analyze the same 10 trials and the results will then be discussed to synchronize search strategy and reduce ambiguities. Both reviewers will independently extract data from all trials, and we will reach conclusions in the following manner:

1. **Same Publication Found by Both Reviewers with Different Earliest Dates:**
   - The publication with the earliest date will be counted.
2. **No Publication Found:**
   - If neither reviewer finds a publication, no publication will be recorded.
3. **Different Publications Found by Each Reviewer:**
   - Both reviewers must agree on the correct publication.
   - In cases where no agreement is reached, a third person will be consulted to decide on the correct publication.

Eligibility criteria for result publications

We will include results publications that correspond to both the following criteria

1. Is a peer-reviewed scientific publication or preprint with >500 words. Doctoral theses, congress abstracts, or other summaries are not counted as results publications unless fulfilling these criteria. Reviews not reporting primary data are not included.

2. Is matched to an eligible clinical trial on all criteria of

a.Study design (intervention model, randomization, masking)

b.Indication/population

c.Intervention/treatment

d.Comparator (if applicable)

Supplementary file

e.The publication's primary outcome measure needs to be listed as an outcome measure in the registration (whether as primary or not). If the publication has several primary outcome measures, the rule applies to at least one of them. If the publication has no primary outcome measure assigned, the rule applies to the first outcome measure mentioned.

Publications will be counted regardless of whether actual enrollment matches planned enrollment since this is often subject to change. Publications that report preliminary analyses for at least one predefined outcome will be counted. Also, publications reporting interim results or fewer study arms are counted (given that the overall study aim is still the same). Publications with reports of several trials combined (without disaggregating between trials) are not included.

Supplementary file

Supplement S3 Global health journals list

Sr no	**Journal Title**	**Journal Description with focus on global health research**	**Include in study?**
1	The Lancet Global Health	Our focus is on disadvantaged populations, be they whole economic regions or marginalised groups within otherwise prosperous nations, with a preference for the following topics: reproductive, maternal, neonatal, child, and adolescent health; infectious diseases, including neglected tropical diseases; non-communicable diseases; mental health; the global health workforce; health systems; surgery; and health policy.	Yes
2	Journal of Global Health	Aims to serve the community of researchers, funding agencies, international organizations, policy-makers and other stakeholders in the field of international health.	Yes
3	Globalization and Health	Provides a platform for research, knowledge sharing and debate on the topic of globalization and its effects on health, both positive and negative.	Yes
4	Annals of Global Health	Aims to advance and disseminate knowledge of global health, promote research and foster the prevention and treatment of disease worldwide.	Yes
5	Pathogens and Global Health	Deals with tropical diseases and medical and veterinary parasitology in their broadest aspects.	Yes
6	Global Public Health	The journal is broad-based and wide-ranging, including work that draws on the environmental health sciences; epidemiology; health policy and management; and the social sciences as applied to public health and medicine.	Yes
7	Global Health Action	Covers the research on public global health: Health information, health determinants, health interventions, environmental change and health, health systems and gender.	Yes

Supplementary file

8	Global Health Promotion	Presents theoretical research and practical papers on experiences, opinions and research leading to applications for health promotion and health education internationally.	Yes
9	BMJ Global Health	Covers all aspects of global health with particular interest in submissions that address the underfunded area of non-communicable diseases.	Yes
10	Global Pediatric Health	Focuses on health issues of children that are common to all regions of the world.	Yes
11	Global Health, Epidemiology, and Genomics	Dedicated to publishing and disseminating research that addresses and increases understanding of global and population health issues through the application of population science, genomics and applied technologies.	Yes
12	Global Health Research and Policy	Aims to rapidly disseminate high quality research to improve regional and global health.	Yes
13	Central Asian Journal of Global Health*	The journal provides a forum for discussion of all aspects of public health, medicine, and global health in Central Asia and around the world.	Yes (excluded later)
14	Clinical Epidemiology and Global Health	Aims to promote clinical epidemiology and global health, with particular emphasis on Indian and Asian countries.	Yes
15	Global Health Journal	Covers all aspects of global health	Yes
16	Global Journal of Health Science	Covers health science, health policy and services, preclinical medicine, clinical medicine, oral medicine, public health and preventive medicine, pharmacy, military and special medicine, medical and related biological research, education, and medical awareness in the public.	Yes
17	Global Mental Health	Aims to cover a broad application of 'the global point of view' of mental health issues.	Yes

Supplementary file

18	International Journal of Travel Medicine and Global Health	Publishes papers in all fields of travel medicine & global health aiming to increase the understanding, diagnosis and treatment of various disorders which can be presented, deteriorated, and healed in a travel. Aims to facilitate the exchange of ideas, hypothesis, techniques and information among all physicians.	Yes
19	PLOS Global Public Health	Publishes diverse research addressing global public health challenges and inequities	Yes
20	Global journal of health science	provide a platform for the global research community to share their findings, insights and views about all aspects of health science	Yes
21	Journal of global health perspectives	Journal of Global Health (JoGH) is a peer-review general medical journal focusing on issues relevant for global health.	Yes

*excluded later as rephrased to "The Central African Journal of Medicine" which is not global health journal per se

Supplementary file

Supplement S4: Global health funders list

**Funder name**	**Description in relation to global health research**	**Online resource to track grants (Yes/No)**	**Clinical trial funder**	**Trial registry number to track funded clinical trial (Yes/No)**	**Link to track grants or grants tracking system**	**Source**
Bill & Melinda Gates Foundation (BMGF)	We provide funding to organizations to achieve measurable impact in the fight against poverty, disease, and inequity around the world.	Yes	Yes	No	https://www.gatesfoundation.org/about/committed-grants	*U.S National Institutes of Health World RePORT
Canadian Institutes of Health Research (CIHR)	As part of its mission, CIHR seeks to foster effective Canadian involvement in international health research that benefits Canadians and the global community.	Yes	Yes	No	https://webapps.cihr-irsc.gc.ca/decisions/p/main.html?lang=en#fq={!tag=programtype2}programtype2%3A%22Randomized%20Controlled%20Trials%22&sort=namesort%20asc&start=0&rows=20	U.S National Institutes of Health World RePORT
European Commission (EC)	The European Commission manages the European Union research and innovation budget and supports research projects that span several years. The european union horizon	Yes	Yes	Yes	https://cordis.europa.eu/search/en	U.S National Institutes of Health World RePORT

Supplementary file

	funding focuses on tackling global challenges					
European & Developing Countries Clinical Trials Partnership (EDCTP)	The EDCTP2 programme supports clinical research, research capacity development and international networking in order to accelerate the clinical development of effective, safe, accessible, suitable and affordable medical interventions for poverty-related infectious diseases affecting sub-Saharan Africa.	Yes	Yes	Yes	https://www.edctp.org/edctp2-project-portal/	U.S National Institutes of Health World RePORT

Supplementary file

German Federal Ministry of Education and Research (BMBF)	The BMBF supports international research activities in sub-Saharan Africa and thus makes a targeted contribution to significantly improving medical care. The focus is on neglected and poverty-related diseases, among other things.<br><br>The fight against the increasing resistance of bacteria and other pathogens to medicines is also a key concern of the G7 countries. This is because widespread antibiotic resistance in particular poses a huge threat to global health. The G7 partners have therefore once again placed the fight against this resistance in the spotlight under the German presidency in 2022. Equally important is joint preparation for	Yes	Yes	No	https://www.gesundheitsforschung-bmbf.de/de/klinische-studien-3384.php	U.S National Institutes of Health World RePORT

Supplementary file

	epidemics or pandemics, which can be triggered by newly emerging pathogens that often jump from the animal kingdom to humans.					
Global Alliance for Chronic Diseases (GACD)	GACD is a global alliance of health research funders representing 80% of public health research funding worldwide	Yes	Yes	No	https://www.gacd.org/research/projects	U.S National Institutes of Health World RePORT
Japan Agency for Medical Research and Development (AMED)	The purpose of this organization is to carry out research and development in the medical field and to improve the environment for it, as well as to provide support, etc., in order to comprehensively and effectively develop the medical field.	Yes	Yes	TBD	https://amedfind.amed.go.jp/amed/index.html	U.S National Institutes of Health World RePORT

Supplementary file

Korea Health Industry Development Institute (KHIDI)	KHIDI supports initiatives to protect the rights and interests of foreign patients, and promotes the convenience of using medical care in Korea so that patients can receive safe and high-quality medical and healthcare services.	No	No	No	https://worldreport.nih.gov/wrapp/#/search?searchId=65f9be981a171a6207c251c3	U.S National Institutes of Health World RePORT
Medical Research Council (MRC)	Launched in April 2018, UK Research and Innovation (UKRI) is a non-departmental public body sponsored by the Department for Science, Innovation and Technology (DSIT).<br>We aim to support UK and low and middle-income country scientists to establish partnerships, participate in global health research opportunities and respond to global challenges in biomedical and health research.	Yes	Yes	No	https://gtr.ukri.org/, https://www.ukri.org/what-we-do/browse-our-areas-of-investment-and-support/?category=mrc&closure_status=open	U.S National Institutes of Health World RePORT

Supplementary file

National Health Medical Research Council (NHMRC)	NHMRC is the key driver of health and medical research in Australia.We engage strategically with international funding agencies and participate in funding schemes that align with the NHMRC International Engagement Strategy 2023-2026.	Yes	Yes	No	https://www.nhmrc.gov.au/funding/data-research	U.S National Institutes of Health World RePORT
National Institutes of Health (NIH)	The Fogarty International Center at the U.S. National Institutes of Health (NIH) is dedicated to advancing the NIH mission by supporting and facilitating global health research conducted by U.S. and international investigators, building partnerships between health research institutions in the U.S. and abroad	Yes	Yes	Yes	https://reporter.nih.gov/search/N2a3NpquYEOspjX9UMAQdg/projects	U.S National Institutes of Health World RePORT
Swedish Research Council (SRC)	We fund research and research infrastructure in all scientific disciplines. We are also advisors to the	Yes	Yes	No	Swecris - Vetenskapsrådet (vr.se)	U.S National Institutes of Health World RePORT

Supplementary file

	Government on research policy issues and work to increase understanding of the long-term societal benefits of research.					
Wellcome Trust	Wellcome is a global charitable foundation established in 1936. That's why we're committed to funding ambitious global research projects that will transform our understanding of life, health, and wellbeing.	Yes	Yes	No	https://wellcome.org/grant-funding/funded-people-and-projects	U.S National Institutes of Health World RePORT
German Research Foundation (DFG)	Collaborative research that involves a diverse range of people from different fields of interest is key to progress in health science – and to achieving our aim of fostering a healthier, happier, world.	Yes	Yes	Yes	https://gepris.dfg.de/gepris/OCTOPUS	Global health funder list by collegeau
German Alliance for Global Health Research	GLOHRA aims to strengthen global health research in Germany by connecting a community of qualified	Yes	Yes	No	https://globalhealth.de/funded-projects.html	Global health funder list by collegeau

Supplementary file

	researchers, funding groundbreaking research projects and scientific events, supporting the next generation of researchers, and enhancing cross-sector collaboration.					
Volkswagen Foundation	Support across borders<br>The Foundation helps to overcome borders – both between academic disciplines and research fields, as well as between countries and cultures.	Yes	Yes	No	https://portal.volkswagenstiftung.de/search/newSearchForm.do	Global health funder list by collegeau

Supplementary file

Supplement S5: Screening studies for PSE reporting manual

Definition of Patient and Stakeholder Engagement (PSE)
Patient and Stakeholder Engagement (PSE) refers to the involvement of patients and other relevant stakeholders (such as caregivers, healthcare providers, patient advocacy groups, policymakers, and the community) in any phase of the clinical study process. This involvement may include contributions to study design, recruitment strategies, data collection, analysis, dissemination of results, or policy integration.

Coding Guidelines
Inclusion Criteria for PSE Reporting

- Any mention of patients or stakeholders being involved in the planning, design, implementation, or dissemination phases of the study.
- Descriptions of how patient or stakeholder input was solicited, used, or valued in the research process.
- References to consultations, focus groups, advisory panels, or committees that include patients or other stakeholders.
- Reports on specific roles or tasks undertaken by patients or stakeholders in the study.
- Mentions of the impact or influence of patient or stakeholder contributions on the study.

Exclusion Criteria

- General mentions of patients or stakeholders without specific reference to their engagement in the research process.
- Solely describing the population characteristics without detailing engagement activities.
- Routine patient consent or standard recruitment processes without additional engagement activities.

Examples of PSE Reporting
Study Design

- "Patients were involved in the development of the study protocol and the selection of outcome measures."
- "A stakeholder advisory panel was consulted to refine the research questions and methodology."

Recruitment

- "Community leaders assisted in developing culturally sensitive recruitment strategies."
- "Patient advocates helped design recruitment materials to ensure they were accessible and understandable."

Data Collection

- "Patients participated in pilot testing the data collection instruments."
- "Stakeholders provided feedback on the data collection process to improve participant experience."

Analysis and Interpretation

- "Patients and caregivers contributed to the interpretation of the study results."
- "Stakeholder input was sought to contextualize the findings and suggest practical implications."

Dissemination

- "Patients co-authored the publication and were involved in presenting the findings at conferences."
- "Stakeholders helped develop dissemination materials targeted at specific communities."

Policy and Practice Integration:

Supplementary file

- "Policy makers were engaged to discuss the potential implications of the study findings for health policy."
- "Stakeholders participated in workshops to translate research findings into practice guidelines."

Coding Procedure:
Read Each Paper Thoroughly: Focus on sections like the introduction, methods, results, discussion, and acknowledgments where PSE might be mentioned.
Highlight Relevant Passages: Use the definition and examples provided to identify and highlight text passages that mention PSE.
Document and Categorize: Record the presence of PSE reporting and categorize the type of engagement (e.g., study design, recruitment, data collection, etc.). Copy-paste PSE mentions in the same excel file.
