# Supplementary material for: Towards monitoring of global health research: an exploratory analysis of transparency and stakeholder engagement
Source: BMJ Open. 2025 Dec 12;15(12):e102010. doi: 10.1136/bmjopen-2025-102010 (PMC12706105; doi:10.1136/bmjopen-2025-102010)
Supplement: Author's manuscript [file bmjopen-2025-102010.draft_revisions.pdf]

BMJ Open is committed to open peer review. As part of this commitment we make the peer review history of every article we publish publicly available.

When an article is published we post the peer reviewers' comments and the authors' responses online. We also post the versions of the paper that were used during peer review. These are the versions that the peer review comments apply to.

The versions of the paper that follow are the versions that were submitted during the peer review process. They are not the versions of record or the final published versions. They should not be cited or distributed as the published version of this manuscript.

BMJ Open is an open access journal and the full, final, typeset and author-corrected version of record of the manuscript is available on our site with no access controls, subscription charges or pay-per-view fees (http://bmjopen.bmj.com).

If you have any questions on BMJ Open's open peer review process please email info.bmjopen@bmj.com

BMJ Open

Towards Monitoring of Global Health Research: An Exploratory Analysis of Transparency and Stakeholder Engagement

Journal:	*BMJ Open*
Manuscript ID	bmjopen-2025-102010
Article Type:	Original research
Date Submitted by the Author:	11-Mar-2025
Complete List of Authors:	Yerunkar, Samruddhi; Charite - Universitatsmedizin Berlin, Department of Psychiatry and Psychotherapy, Charité ; Berlin Institute of Health at Charite QUEST Center for Responsible Research<br>Hildebrand, Nicole; Charité - Universitätsmedizin Berlin, Department of Psychiatry and Psychotherapy, Charité<br>Strech, Daniel; Berlin Institute of Health at Charite QUEST Center for Responsible Research
Keywords:	Tuberculosis < INFECTIOUS DISEASES, Clinical trials < THERAPEUTICS, Puerperal Disorders, Depression & mood disorders < PSYCHIATRY, Postpartum Period

SCHOLARONE™
Manuscripts

I, the Submitting Author has the right to grant and does grant on behalf of all authors of the Work (as defined in the below author licence), an exclusive licence and/or a non-exclusive licence for contributions from authors who are: i) UK Crown employees; ii) where BMJ has agreed a CC-BY licence shall apply, and/or iii) in accordance with the terms applicable for US Federal Government officers or employees acting as part of their official duties; on a worldwide, perpetual, irrevocable, royalty-free basis to BMJ Publishing Group Ltd ("BMJ") its licensees and where the relevant Journal is co-owned by BMJ to the co-owners of the Journal, to publish the Work in this journal and any other BMJ products and to exploit all rights, as set out in our licence.

The Submitting Author accepts and understands that any supply made under these terms is made by BMJ to the Submitting Author unless you are acting as an employee on behalf of your employer or a postgraduate student of an affiliated institution which is paying any applicable article publishing charge ("APC") for Open Access articles. Where the Submitting Author wishes to make the Work available on an Open Access basis (and intends to pay the relevant APC), the terms of reuse of such Open Access shall be governed by a Creative Commons licence – details of these licences and which Creative Commons licence will apply to this Work are set out in our licence referred to above.

Other than as permitted in any relevant BMJ Author's Self Archiving Policies, I confirm this Work has not been accepted for publication elsewhere, is not being considered for publication elsewhere and does not duplicate material already published. I confirm all authors consent to publication of this Work and authorise the granting of this licence.

For peer review only

Towards Monitoring of Global Health Research: An Exploratory Analysis of Transparency and Stakeholder Engagement

Samruddhi Yerunkar (SSY)[1 2*], Nicole Hildebrand (NH)[2], Daniel Strech (DS)[1]
[1]QUEST Center for Responsible Research, Berlin Institute of Health (BIH) at Charité – Universitätsmedizin Berlin, Berlin, Germany
[2]Department of Psychiatry and Psychotherapy, Charité - Universitätsmedizin Berlin, Campus Benjamin Franklin, Berlin, Germany

*Corresponding author: Samruddhi Yerunkar
Address: Berlin Institute of Health (BIH) at Charité – Universitätsmedizin Berlin, Berlin, Germany
Email: samruddhi.yerunkar@charite.de

For peer review only

Abstract

Introduction

Global health research (GHR) requires transparent practices and stakeholder engagement to maximize impact. While monitoring systems exist for clinical research transparency in high-income countries, there is limited systematic assessment of these practices in global health research. This study evaluated methods for monitoring GHR transparency and engagement practices using established indicators.

Methods

We analyzed three samples: (1) 200 interventional trials from ClinicalTrials.gov (2008-2019) focused on tuberculosis and maternal health, with two-thirds from low-and-middle-income countries; (2) 200 trial publications from global health journals (2011-2023); and (3) research outputs from global health funder websites. We assessed registration timing, result reporting, open access status, and stakeholder engagement using standardized indicators.

Results

Among registry trials, 37% were prospectively registered, 65% published results in journals, and 15% reported summary results in ClinicalTrials.gov. Only 34% reported results in any format within 24 months of completion. For journal publications, 72% were freely accessible, and among a subsample of 100 articles, 23% included stakeholder engagement statements. The funder website sample yielded insufficient metadata for systematic analysis.

Conclusion

Our findings demonstrate that monitoring GHR transparency and engagement is feasible using trial registries and journal publications, though funder websites currently lack adequate tracking data. While open access rates are encouraging, timely result reporting and stakeholder engagement documentation need improvement. These results highlight opportunities for developing GHR-specific monitoring approaches through collaborative efforts among global stakeholders.

Funding

Berlin University Alliance

Strengths and Limitations of this study

This study introduces a methodology suitable for assessing GHR studies with transparency and engagement indicators. We sourced data from trial registries, global health journals, and funder websites, providing accompanying code and data to facilitate reproducibility for other researchers in the field. Our findings offer empirical data while highlighting the need for expanded collaboration to refine indicators and methodologies, enhancing the contextual relevance of GHR monitoring.

This study utilizes only a sample of studies for exploratory analysis, which limits the generalizability of the findings. Similarly, the trial registry sampling strategy required careful selection of conditions, which could introduce bias or limit the scope of research captured. Additionally, the journal-based approach is limited by the need to rely on global health journals to represent all GHR research.

For peer review only

1. Introduction

Global health research (GHR) is a collaborative, transnational research approach aimed at promoting health equity for all (1). The field addresses major health challenges that affect large populations worldwide, often focusing on severe conditions with substantial impact on mortality and quality of life, particularly in resource-limited settings. Recent surges in GHR activities, especially spurred by the COVID-19 pandemic, have highlighted both the field's potential and its challenges in delivering timely evidence for decision-making.

Research transparency and community engagement are crucial elements across all fields of health research. In GHR, these practices take on additional dimensions due to the field's unique characteristics. For instance, many global health conditions affect substantial populations across different regions and cultural contexts, making rapid knowledge translation particularly important. Limited research infrastructure in many settings and historical power imbalances between researchers and communities further emphasize the need for transparent research practices and meaningful stakeholder engagement.

The timely dissemination of research findings through transparent practices such as prospective registration, comprehensive results reporting, and open-access publication is essential for evidence-based healthcare globally. In GHR, barriers to accessing research findings can be especially problematic - paywalled publications may be inaccessible to healthcare providers, researchers, and patients in low-resource settings, potentially widening rather than reducing health inequities. Similarly, the transnational and cross-cultural nature of GHR makes genuine patient and stakeholder engagement (PSE) particularly relevant for ensuring research relevance, cultural appropriateness, and eventual implementation.

Historically, global health monitoring was primarily led by major inter-governmental agencies. However, academic research has increasingly complemented these efforts, enhancing the methods and rigour of global health measurement to support better decision-making by agencies, governments, and donors (2). While significant efforts have been made to monitor trial transparency and engagement in Europe and North America (3-7), gaps remain in assessing these practices on a global scale (8). For instance, the EU TrialsTrackers (http://eu.trialstracker.net/) specifically monitor whether summary results are posted on the EU clinical trials register and national-level dashboards have also been developed, providing a detailed breakdown of practices and helping stakeholders identify and address areas needing improvement (7, 9). Similar GHR monitors can help assess the current landscape, highlight actionable areas, and guide stakeholders—including patients, researchers, physicians, and global leaders—in making informed decisions.

Improved global health monitoring further requires new technologies and methods, as well as established norms and standards to facilitate global reporting (10). To build an effective monitoring system for global health, it is important to first define what constitutes global health studies and then assess with appropriate indicators. We explored three methods for generating a sample of global health studies: (1) clinical trial registries focusing on global health conditions or diseases affecting populations globally, (2) publications from global health journals, and (3) research outputs mentioned on global health funders' websites. Using indicators established in previous studies, we assessed a sample of these studies to provide empirical data. We aim to demonstrate how past methodologies for monitoring clinical research can be applied to GHR, highlighting opportunities and challenges to enhance the implementation of monitoring systems in the GHR.

2. Methods

2.1. Sampling Strategy

We explored three complementary approaches to identify global health research studies: (1) clinical trial registries, using selected global health conditions as proxy, (2) publications from journals with an explicit global health focus, and (3) research outputs listed on global health funder websites. Each source offered distinct advantages and challenges for identifying and tracking global health studies. Below, we describe the methodology and purpose.

For peer review only

2.1.1 **Clinical Trial Registry with a disease-based approach**:

We systematically searched trial registry of ClinicalTrials.gov for interventional trials with start date between January 1, 2008 to completion date March 31, 2019. This eligibility requirement was based on the mandate for clinical trial registration in registries under both US laws and journal policies following ICMJE guidelines (11) passed on January 1, 2008. For our analysis, we focused on two health conditions known to have significant global burdens: maternal health issues (including postpartum depression, maternal sepsis, and maternal anaemia) and tuberculosis. These conditions were chosen based on their significant global health burden and their relevance to our ongoing larger research project, where one of our team members has a specific interest in maternal health, particularly postpartum conditions. We excluded international multicentric trials, observational studies, and those with missing location or site information, as well as withdrawn studies. Trial locations were categorized as high-income countries (HIC) or low-to-middle-income countries (LMIC) according to the World Bank country classification (12). For our exploratory analysis, we randomly selected 100 trials per condition. To address the imbalance, with more than half of the trials originating from HICs, we ensured that two-thirds of the selected trials were from LMICs. This approach ensured equitable representation and also addressed the historical disparity in research quality assessments between HICs and LMICs. In this sample, we aim to demonstrate the feasibility of evaluating registration practices and the timeliness of result reporting by examining both summary results and publications from a sample of clinical trials within a global health context.

To locate trial publications, two independent reviewers (NH and SSY), followed a predefined search manual to identify the earliest publications of trial results (see search manual). Searches were conducted in both the registry and Google, using trial IDs and relevant terms. Eligible publications included peer-reviewed articles or preprints with over 500 words, matched to the trial by design, population, intervention, and comparator (if applicable). Additionally, the publication's primary outcome measure (or first mentioned outcome) had to be listed as an outcome measure in the registration, whether classified as primary or not.

2.1.2 **Global Health Journals**:

Our second approach focussed on generating a sample of global health studies from peer-reviewed journals specializing in global health. We identified 20 journals that either had 'global health' or 'international health' in their title (see list of journals). Using the Cochrane Highly Sensitive Search Strategy (Box 3b strategy), we located randomized trials published after January 1st, 2011, in PubMed and cross-referenced these results with our selected journals. Including trial publications post-2011 allowed a three-year window for dissemination of results, following the ICMJE mandate for prospective trial registration, effective from 2008. Our search yielded 1,125 records of trials published after 2011. An author (SSY) screened a random sample (n = 300), categorizing them as primary reports of trial results or secondary analyses by the original or different trial investigators. For analysis, we included the first 200 primary reports and secondary analyses by same investigators (see Supplementary Figure 1 flowchart). This sample was used to evaluate how trial results are reported in GHR, examining adherence inclusion of data sharing, and community engagement statements.

2.1.3 **Global Health Funder website**:

The third method aimed to explore the use of global health funder websites as potential sources for generating global health studies. We investigated funder websites to determine if they provided sufficient meta-data, such as trial registration numbers and registry links, to allow for the tracking of funded clinical trials and to assess transparency. This involved searching World RePORT (13), a database for tracking funding data for some largest biomedical funders, as well as a list of global health funders compiled by colleagues for a prior project. Our search revealed 20 global health funders and 1 of them had explicit GH programs with available funding meta-data, sufficient to track funded clinical trials (see list of global health funders).

We focused on the Fogarty International Center (FIC) at the National Institutes of Health (NIH), which supports global health research. Using the NIH RePORTER tool, we identified a list of 73 clinical trials and 9515 publications supported by 331 core projects. However, after applying the inclusion criterion with regard to date, the number of relevant trials was very limited, leading us to exclude this sample from our analysis.

For peer review only

Table 1: Established indicators used for assessing transparency and stakeholder engagement in global health research samples

Indicator	Details	Assessment	Impact
**Follow-up of clinical trials from clinical trial registry with a disease-based approach**			
Time to registration	Before any clinical trial is initiated its details must be registered in a publicly available, free-to-access, searchable clinical trial registry complying with WHO's international agreed standards[1].	Automated check by comparing the trial registration date with the trial start date. We report on<br>1) before the trial starts (prospective registration),<br>2) within 60 days after the trial starts (early registration),<br>3) after 60 days (late registration)	Transparency, unbiased reporting
Result reporting	Results can be reported in the following way: Published articles in a peer-reviewed journal (24 months from trial completion); Trial results uploaded in the trial registry (12 months from trial completion)[1].	Automated check for summary result reporting in the registry and manual search[2] for published articles. We report on<br>1) Summary result (SR) in registry<br>2) Journal publication<br>3) SR or journal publication within 1 year after trial completion<br>4) SR or journal publication within 2 years after trial completion<br>5) SR or journal publication within 4 years after trial completion	Transparency, informed decision making
Open Access	Publications describing clinical trial results should be open-access[1].	Automated check for publication status (open access) for manually searching research publications of clinical trials.	Transparency, accessibility
**Analysis of clinical trial result publication in global health journals**			
Community Engagement/Patient Stakeholder Reporting	Involvement of patients and other relevant stakeholders (such as caregivers, healthcare providers, patient advocacy groups, policymakers, and the community) in any phase of the clinical study process.	Manual review of publications for stakeholder involvement statements.	Practical relevance, inclusivity
Open Access	Publications describing clinical trial results should be open-access[1].	Automated check for publication status (open access)	Transparency, accessibility
Data sharing statement	Data sharing, archiving, depositions, and accessibility of data in journal articles with or without request	Automated check for declarations on data sharing and availability.	Transparency, reproducibility
Code sharing statement	Support sharing of health research datasets whenever appropriate	Automated check for open code statements.	Transparency, reproducibility

[1] Text referenced from WHO Joint statement on public disclosure of results from clinical trials (https://www.who.int/news/item/18-05-2017-joint-statement-on-registration).
[2] Manual searches are done according to the search manual for locating result publications (https://osf.io/9hktj).

2.2. Software

We used the aactr R package (14) to assess registration and summary result reporting in registry of trials. The R packages oddpub (15) and roadoi (16) were used to assess availability statements and open access status respectively in trial publications. Map was created using the Free and Open Source QGIS (17). Data cleaning steps and statistical analyses were performed using R (18). All analysis scripts are available under an open license on GitHub https://github.com/quest-bih/prodigy.

For peer review only

3. Results

This section provides an independent analysis of two samples: the first focuses on interventional trials from ClinicalTrials.gov, while the second examines trial result publications from global health journals. Data from the third funder website sample was limited and not analyzed.

3.1 Follow-up of clinical trials from Clinical trial registry

We included data from 200 interventional trials (2009 – 2019) with 2/3rd representation from LMIC, enrolling 115,929 participants. Dominant trial characteristics were small sample size (<100) and open labelled (Table 1). The most common trial locations were the United States (n = 25), South Africa (n = 24), and China (n = 23) (Figure 1).

Table 2 Characteristics of included clinical trials

Trial characteristic[1]	Tuberculosis (n =100)	Maternal Health Conditions (n = 100)	All trials, N= 200
**Recruitment status**			
Completed	79 (79)	76 (76)	155 (77.5)
Terminated	2 (2)	8 (8)	10 (5)
Unknown status	19 (19)	16 (16)	35 (17.5)
**Enrolment**			
1- 100	49 (49)	40 (40)	89 (44.5)
100 - 500	32 (32)	44 (44)	76 (38)
>500	19 (19)	16 (16)	35 (17.5)
**Masking** (missing, n =1)			
Open label	62 (62)	53 (53)	115 (57.5)
Any masking	37 (37)	47 (47)	84 (42)
**Design** (missing, n =29)			
Non-randomized	10 (10)	15 (15)	25 (12.5)
Randomized	76 (76)	70 (70)	146 (73)
**Phase**			
I	19 (19)	5 (5)	24 (12)
I-II	3 (3)	1 (1)	4 (2)

For peer review only

II	21 (21)	14 (14)	35 (17.5)
II-III	2 (2)	2 (2)	4 (2)
III	9 (9)	13 (13)	22 (11)
IV	11 (11)	24 (24)	35 (17.5)
Not applicable	35 (35)	41 (41)	76 (38)

[1]Total count may differ between categories because of missing data (number missing shown if >0).

Trial registration and result reporting

Of the 200 trials, 74 (37%) were registered prospectively. Regarding result reporting, from all 200 trials that were completed until 2019 or earlier and thus had 4 or more years to report results, 129 trials (65%) had results published in journals, and 30 (15%) reported summary results in registries. The number of trials that reported any results (either via journals or summary results) within one, two, or four years after completion was 29 trials (15%), 67 trials (34%) and 116 (58%) (Table 3).

For journal result publications identified using a search manual, the reviewer independently located relevant publications with an interrater reliability of 83%. When different publications were selected, 100% agreement was reached on selecting the earliest matching publication. All matched publications were peer-reviewed articles, with majority – specifically, 60% (78 /129) - linked in the registry.

Table 3 Registration and result reporting of included clinical trials

Indicators	Result (n, %)
	All trials, N= 200
**Time to registration**	
Prospective registration	74 (37)
Within 60 days after trial start	50 (25)
>60 days after trial start	76 (38)
**Result reporting**	
Journal Publication	129 (65)
Summary result (SR) in registry	30 (15)
Result reporting within (SR or Journal) 1 year after completion	29 (14.5)
Results reporting (SR or Journal) within 2 years after completion	67 (34)
Result reporting (SR or Journal) within 4 years after completion	116 (58)
Open Access (n = 125[1])<br>Gold:22, Green: 20, Hybrid: 13, Bronze:10, Closed: 15, NA: 45	65 (52)

[1] For all 129 journal publications, we could find DOI for 125 articles

3.2 Analysis of trial results in publications from GH journals

Of the 200 trial publications in global health journals between 2011 to 2023, 72% (144) were Gold Open Access (freely accessible), 22.5% (45) were Hybrid Open Access (partially accessible), and 1.5% (3) were closed access. We were able to download 127 of the 200 PDFs to assess data and code availability. Among these, 59% (75) included a data-sharing statement, often specifying availability upon request, and only 0.8% (1) included a code-sharing statement. A random sample of 100 articles was reviewed for stakeholder engagement statements using a predefined protocol (see search manual) with 23% (23) reporting relevant content (see extracted statements).

Table 2: Transparency and community engagement in included trial result publication

Indicators	Result (n, %)
Open Access (n = 200)	197 (99)

For peer review only

Gold: 144, Green: 8, Hybrid:45, Closed:3,	
Data sharing statement including 'available upon request' (n = 127[1])	75 (59)
Code sharing statement (n = 127[1])	1 (0.8)
Community/Stakeholder engagement statement (n = 100[2])	23 (23)

[1]For all 200 we had 127 PDFs, [2]Checked for a random sample of 100 publications

4. Discussion

This study aimed to explore approaches for monitoring transparency and patient/stakeholder engagement in Global Health Research (GHR) while providing empirical data on the current state of these practices. We tested three different sampling strategies and found that trial registries focusing on global health conditions and global health journals provide viable data sources for monitoring, while funder websites currently lack sufficient metadata for systematic tracking.

Among 200 registered trials, 37% were prospectively registered, and 65% had published results in journals, though only 15% reported summary results. Within 24 months of trial completion, only 34% reported their result in any format and thus demonstrated timely results reporting. These findings on timely results reporting are comparable to previous studies examining clinical trial transparency in other contexts, such as German university medical centers (43%) (6), Nordic countries (52%) (5), and US academic medical centers (36%) (19). Patient/Stakeholder engagement was mentioned in 23% of publications, providing a baseline for more detailed future analyses of engagement practices in GHR.

Our methodological approach had several strengths and limitations. The disease-based sampling strategy using trial registries proved effective for identifying GHR studies but required careful selection of conditions. Among identified publications, 60% were properly linked to their registry entries, indicating moderate but improvable compliance with WHO recommendations for result traceability. The journal-based approach successfully identified GHR publications but is limited by the need to rely on "global health journals" to represent global health research. The funder-based approach highlighted current gaps in tracking systems that need to be addressed to enable comprehensive monitoring. A key methodological challenge was ensuring balanced representation between high- and low-income countries. Our approach of oversampling LMIC-based studies (2/3 of trial registry sample) provides one potential solution, though optimal sampling strategies for GHR monitoring should be further discussed with stakeholders.

While the overall rates of timely reporting are similar to other contexts, timely results reporting is particularly important in GHR given that many global health conditions represent a high global burden of disease (GBD). Although it would be problematic to claim that timely reporting is more important in some research fields than others, the often substantial disease burden addressed in GHR emphasizes the need for rapid knowledge translation to inform evidence-based healthcare decisions. The observed high rate of open-access publications (99%) in our sample is particularly encouraging, as paywalled research results would disproportionally affect healthcare providers and researchers in low-resource settings. This rate markedly exceeds the average of 28% open access found across scholarly literature. While this high rate may partly reflect our sampling of global health journals with established open-access policies and increasing pressure on global health researchers to publish transparently, it nevertheless demonstrates substantial progress in making GHR findings widely accessible.

The increasing adoption of institutional monitoring tools, as demonstrated by Franzen et al. (7), could help track and improve transparency in GHR. Such tools should incorporate core open science practices recently identified through expert consensus (20) while considering GHR-specific needs. The baseline measurement of patient/stakeholder engagement reporting provides important insights for future development. Given that GHR often involves transnational and cross-cultural research contexts, meaningful engagement of local communities and stakeholders is particularly crucial. However, our finding that only 23% of publications mentioned engagement activities suggests room for improvement. As demonstrated by Weschke et al. (21), more detailed analyses of engagement reporting can reveal important insights about the quality and impact of these practices.

In our analysis, we assessed only a sample of studies to provide empirical data on how GHR can be monitored. However, global health research is inherently collaborative, requiring active input from researchers and

For peer review only

stakeholders. Future efforts should identify additional indicators for monitoring, address challenges in informing researchers about their performance in transparency and engagement, and expand on our findings using new samples and methodologies. Continued monitoring can drive improved practices, fostering more transparent and inclusive GHR to support evidence-based global health advancements.

5. Data sharing

All the code for this study is available via open-source licensing on GitHub (https://github.com/quest-bih/prodigy). All underlying raw and processed datasets for this study are available for download from the Open Science Framework (https://osf.io/hqdns/).

6. Declaration of interests

We declare no competing interests.

7. Patient and public involvement

Patients and/or the public were not involved in the design, or conduct, or reporting, or dissemination plans of this research.

8. Patient consent for publication

Not applicable.

9. Ethics approval

Not applicable.

10. Acknowledgments

This study is part of the PRODIGY project and we would like to thank Malek Bajbouj, Isabel Dziobek, Eric Hahn, Thi Minh Tam Ta, María Jose Lobeda-Garzón as well as Sarah Weschke, Vladislav Nachev and Delwen Franzen who contributed their ideas and knowledge to make this study possible.

11. Author contributions

Conceptualization: SSY, DS

Formal analysis: SSY

Funding acquisition: DS

Investigation: SSY, NH

Methodology: SSY, DS

Project administration: SSY

Supervision: DS

Writing – original draft: SSY

Writing – review & editing: SSY, NH, DS

12. Funding

For peer review only

This work was funded by the Berlin University Alliance (BUA). DS received funding for a work package within the PRODIGY project. The funder had no role in the study design, data collection and analysis, decision to publish, or preparation of the manuscript.

13. References

1. Koplan JP, Bond TC, Merson MH, Reddy KS, Rodriguez MH, Sewankambo NK, et al. Towards a common definition of global health. Lancet. 2009;373(9679):1993-5. https://doi.org/10.1016/S0140-6736(09)60332-9
2. Park JJH, Mogg R, Smith GE, Nakimuli-Mpungu E, Jehan F, Rayner CR, et al. How COVID-19 has fundamentally changed clinical research in global health. Lancet Glob Health. 2021;9(5):e711-e20. https://doi.org/10.1016/S2214-109X(20)30542-8
3. Macleod MR, Michie S, Roberts I, Dirnagl U, Chalmers I, Ioannidis JP, et al. Biomedical research: increasing value, reducing waste. Lancet. 2014;383(9912):101-4. https://doi.org/10.1016/S0140-6736(13)62329-6
4. Strzebonska K, Wasylewski MT, Zaborowska L, Riedel N, Wieschowski S, Strech D, et al. Results dissemination of registered clinical trials across Polish academic institutions: a cross-sectional analysis. BMJ Open. 2020;10(1):e034666. https://doi.org/10.1136/bmjopen-2019-034666
5. Nilsonne G, Wieschowski S, DeVito NJ, Salholz-Hillel M, Bruckner T, Klas K, et al. Results reporting for clinical trials led by medical universities and university hospitals in the Nordic countries was often missing or delayed. medRxiv. 2024.
6. Wieschowski S, Riedel N, Wollmann K, Kahrass H, Muller-Ohlraun S, Schurmann C, et al. Result dissemination from clinical trials conducted at German university medical centers was delayed and incomplete. J Clin Epidemiol. 2019;115:37-45. https://doi.org/10.1016/j.jclinepi.2019.06.002
7. Franzen DL, Carlisle BG, Salholz-Hillel M, Riedel N, Strech D. Institutional dashboards on clinical trial transparency for University Medical Centers: A case study. PLoS Med. 2023;20(3):e1004175. https://doi.org/10.1371/journal.pmed.1004175
8. Tijdink JK, Horbach S, Nuijten MB, O'Neill G. Towards a Research Agenda for Promoting Responsible Research Practices. J Empir Res Hum Res Ethics. 2021;16(4):450-60. https://doi.org/10.1177/15562646211018916
9. Jeangirard E. Monitoring Open Access at a national level: French case study. ELPUB 2019 23rd edition of the International Conference on Electronic Publishing; 2019-06-02; Marseille, France: African Society in Digital Science; 2019.
10. Murray CJ, Lopez AD, Wibulpolprasert S. Monitoring global health: time for new solutions. BMJ. 2004;329(7474):1096-100. https://doi.org/10.1136/bmj.329.7474.1096
11. International Committe of Medical Journal Editors ICMJE. [Available from: https://www.icmje.org/about-icmje/faqs/clinical-trials-registration/.
12. 2007 WB. Data & Statistics. Country Groups. Washington, DC: World Bank. [Available from: http://web.worldbank.org/WBSITE/EXTERNAL/DATASTATISTICS/0,,contentMDK:20421402~pagePK:64133150~piPK:64133175~theSitePK:239419,00.html#Low_income.
13. World RePORT [Internet]. [cited 10th February 2024]. Available from: https://worldreport.nih.gov/wrapp/#/search?searchId=664f6f671a171a6207c2dabd.
14. Salholz-Hillel M. aactr R package GitHub [Available from: https://github.com/maia-sh/aactr].
15. Riedel N, Kip, M. and Bobrov, E. ODDPub – a Text-Mining Algorithm to Detect Data Sharing in Biomedical Publications. Data Science Journal. 2020. https://doi.org/https://doi.org/10.5334/dsj-2020-042.
16. Jahn N. roadoi: Find Free Versions of Scholarly Publications via Unpaywall 2024 [Available from: https://docs.ropensci.org/roadoi/].
17. QGIS Development Team. QGIS Geographic Information System,. QGIS Association; 2024.
18. R Core Team. R: A language and environment for statistical computing. Vienna, Austria. p. R Foundation for Statistical Computing 2022.

For peer review only

19. Chen R, Desai NR, Ross JS, Zhang W, Chau KH, Wayda B, et al. Publication and reporting of clinical trial results: cross sectional analysis across academic medical centers. BMJ. 2016;352:i637. https://doi.org/10.1136/bmj.i637

20. Cobey KD, Haustein S, Brehaut J, Dirnagl U, Franzen DL, Hemkens LG, et al. Community consensus on core open science practices to monitor in biomedicine. PLoS Biol. 2023;21(1):e3001949. https://doi.org/10.1371/journal.pbio.3001949

21. Weschke S, Franzen DL, Sierawska AK, Bonde LS, Strech D, Schorr SG. Reporting of patient involvement: a mixed-methods analysis of current practice in health research publications using a targeted search strategy. BMJ Open. 2023;13(1):e064170. https://doi.org/10.1136/bmjopen-2022-064170

For peer review only

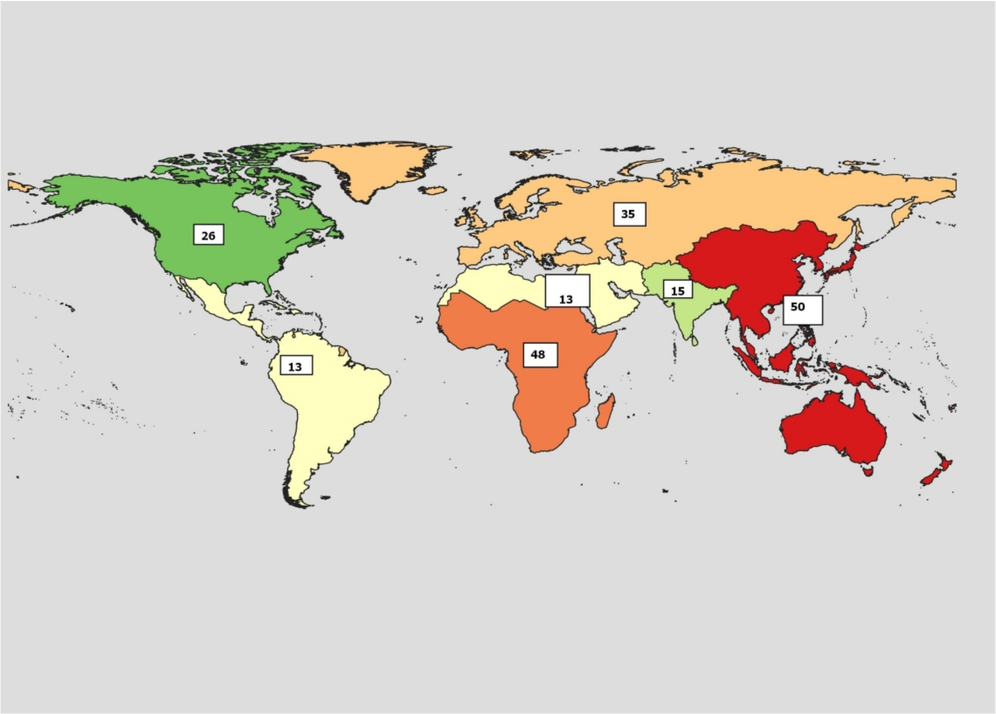

Figure 1 Geographic distribution of included clinical trials

256x183mm (300 x 300 DPI)

Supplementary file 1

Supplementary Method details

1. Trial Registry using Global Health Condition

On 13th April 2024, using the ClinicalTrials.gov web front (https://clinicaltrials.gov/), we searched studies by Condition/disease (MeSH terms) in the following way:

Condition	N
Postpartum Depression (also searched for **Depressive Disorders**, **Puerperal**, and **Post Natal Depression**. See Search Details)	450
Maternal Sepsis (also searched for **Depressive Disorders**, **Puerperal**, and **Post Natal Depression**. See Search Details)	1403
Maternal Anemia (also searched for **Anemia of pregnancy**, **Pregnant**, **Anemia during pregnancy** and more. See Search Details)	437
Tuberculosis (See Search Details)	1391

2. Global Health Journal

On 13th April 2024, using the Cochrane Highly Sensitive Search Strategy (Box 3b strategy), we identified randomized trials in PubMed and cross-referenced these results with articles from 20 global health journals.

Box 3. b Cochrane Highly Sensitive Search Strategy for identifying randomized trials in MEDLINE: sensitivity- and precision-maximizing version (2008 revision); PubMed format

#1 randomized controlled trial [pt]

#2 controlled clinical trial [pt]

#3 randomized [tiab]

#4 placebo [tiab]

#5 clinical trials as topic [mesh:noexp]

#6 randomly [tiab]

#7 trial [ti]

#8 #1 OR #2 OR #3 OR #4 OR #5 OR #6 OR #7

#9 animals [mh] NOT humans [mh]

#10 #8 NOT #9

PubMed search syntax:
[pt] denotes a Publication Type term;
[tiab] denotes a word in the title or abstract;
[sh] denotes a subheading;
[mh] denotes a Medical Subject Heading (MeSH) term 'exploded';
[mesh:noexp] denotes a Medical Subject Heading (MeSH) term not 'exploded';
[ti] denotes a word in the title.

For peer review only

Supplementary file 1

PubMed terms used to extract articles

((((("The Lancet. Global health"[Journal]) OR ("Journal of global health"[Journal])) OR ("Globalization and health"[Journal])) OR ("Annals of global health"[Journal])) OR ("pathogens and global health"[Journal])) OR ("Global public health"[Journal])) OR ("Global health action"[Journal])) OR ("Global health promotion"[Journal])) OR ("BMJ global health"[Journal])) OR ("Global pediatric health"[Journal])) OR ("Global health, epidemiology and genomics"[Journal])) OR ("Global health research and policy"[Journal])) OR ("The Central African journal of medicine"[Journal])) OR ("Clinical epidemiology and global health"[Journal])) OR ("Global journal of health science"[Journal])) OR ("Global mental health (Cambridge, England)"[Journal])) OR ("international journal of travel medicine and global health"[Journal])) OR ("plos global public health"[Journal])) OR ("Global journal of health science"[Journal])) OR ("journal of global health perspectives"[Journal])) OR ("Global health journal (Amsterdam, Netherlands)"[Journal])) and (((randomized controlled trial[Publication Type]) OR (controlled clinical trial[Publication Type]) OR (randomized[Title/Abstract]) OR (placebo[Title/Abstract]) OR (clinical trials as topic[mesh:noexp]) OR (randomly[Title/Abstract]) OR (randomly[Title/Abstract]) OR (trial[Title])) NOT (animals [mh] NOT humans [mh]))) NOT review [pt]) AND ((english[Filter]) AND (2011:2024[pdat]))) Filters: English, from 2011 – 2024

Note: We additionally excluded articles (n = 9) from “The Central African journal of medicine” journal, which was not explicitly a global health journal. Please see “13-4-2024-global-health-article” for details.

For peer review only

Supplementary file 1

Supplementary Figure 1 Global Health Journal Analysis Flowchart

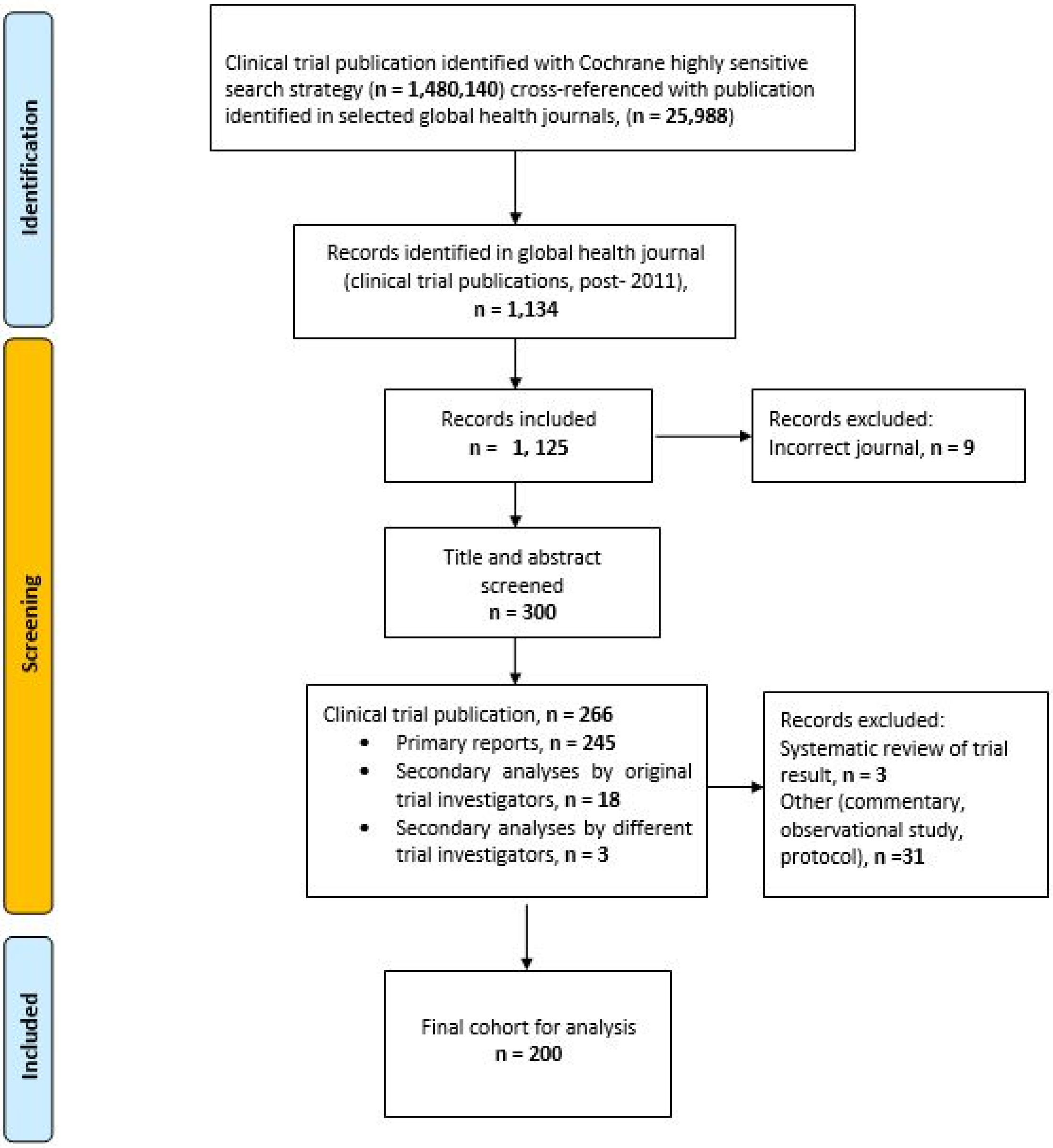

Supplementary file 1

3. Global Health Funder

On 13th April 2024, using the NIH RePORTER tool's 'Advanced Project Search' (https://reporter.nih.gov/advanced-search) and selecting the Funding tab, with Agency/Institute/Center set to "John E. Fogarty International Center for Advanced Study in the Health Sciences (FIC)," the search results included:

- 73 clinical trials,
- 9,515 publications,
- Supported by 331 core projects.

For peer review only

BMJ Open

Towards Monitoring of Global Health Research: An Exploratory Analysis of Transparency and Stakeholder Engagement

Journal:	*BMJ Open*
Manuscript ID	bmjopen-2025-102010.R1
Article Type:	Original research
Date Submitted by the Author:	04-Nov-2025
Complete List of Authors:	Yerunkar, Samruddhi; Charite - Universitatsmedizin Berlin, Department of Psychiatry and Psychotherapy, Charité ; Berlin Institute of Health at Charite QUEST Center for Responsible Research<br>Hildebrand, Nicole; Charité - Universitätsmedizin Berlin, Department of Psychiatry and Psychotherapy, Charité<br>Strech, Daniel; Charité Universitätsmedizin Berlin,
**Primary Subject Heading**:	Global health
Secondary Subject Heading:	Ethics, Research methods
Keywords:	Tuberculosis < INFECTIOUS DISEASES, Clinical trials < THERAPEUTICS, Puerperal Disorders, Depression & mood disorders < PSYCHIATRY, Postpartum Period

SCHOLARONE™
Manuscripts

I, the Submitting Author has the right to grant and does grant on behalf of all authors of the Work (as defined in the below author licence), an exclusive licence and/or a non-exclusive licence for contributions from authors who are: i) UK Crown employees; ii) where BMJ has agreed a CC-BY licence shall apply, and/or iii) in accordance with the terms applicable for US Federal Government officers or employees acting as part of their official duties; on a worldwide, perpetual, irrevocable, royalty-free basis to BMJ Publishing Group Ltd ("BMJ") its licensees and where the relevant Journal is co-owned by BMJ to the co-owners of the Journal, to publish the Work in this journal and any other BMJ products and to exploit all rights, as set out in our licence.

The Submitting Author accepts and understands that any supply made under these terms is made by BMJ to the Submitting Author unless you are acting as an employee on behalf of your employer or a postgraduate student of an affiliated institution which is paying any applicable article publishing charge ("APC") for Open Access articles. Where the Submitting Author wishes to make the Work available on an Open Access basis (and intends to pay the relevant APC), the terms of reuse of such Open Access shall be governed by a Creative Commons licence – details of these licences and which Creative Commons licence will apply to this Work are set out in our licence referred to above.

Other than as permitted in any relevant BMJ Author's Self Archiving Policies, I confirm this Work has not been accepted for publication elsewhere, is not being considered for publication elsewhere and does not duplicate material already published. I confirm all authors consent to publication of this Work and authorise the granting of this licence.

For peer review only

Towards Monitoring of Global Health Research: An Exploratory Analysis of Transparency and Stakeholder Engagement

Samruddhi Suresh Yerunkar (SSY)[1 2*], Nicole Hildebrand (NH)[2], Daniel Strech (DS)[1]
[1]QUEST Center for Responsible Research, Berlin Institute of Health (BIH) at Charité – Universitätsmedizin Berlin, Berlin, Germany
[2]Department of Psychiatry and Psychotherapy, Charité - Universitätsmedizin Berlin, Campus Benjamin Franklin, Berlin, Germany

*Corresponding author: Samruddhi Suresh Yerunkar
Address: Berlin Institute of Health (BIH) at Charité – Universitätsmedizin Berlin, Berlin, Germany
Email: samruddhi.yerunkar@charite.de

For peer review only

Abstract

Objectives: Monitoring systems exist for clinical research transparency in high-income countries, but systematic assessment of these practices in global health research (GHR) is limited. We evaluated methods for monitoring GHR transparency and engagement.

Design: Cross-sectional study.

Data sources: Three sources were used: (a) ClinicalTrials.gov, (b) publications from 20 journals with "international" or "global" in the title, and (c) outputs from selected GH funder websites.

Eligibility criteria: From ClinicalTrials.gov, we selected 200 interventional trials on maternal health and tuberculosis (2008-2019), ensuring two-thirds were from low-and middle-income countries (LMIC). From journals, we included 200 trial publications (2011-2023). From funder websites, we included outputs with sufficient metadata to track trial registration and reporting.

Data extraction and synthesis: Trials were extracted independently by two reviewers for result publications; journal articles were screened to confirm whether they reported trial results. Across all sources, we assessed registration timing, result reporting, open access, and stakeholder engagement.

Results: For 200 trials, 37% were prospectively registered, 65% published results in journals, and 15% reported summary results in ClinicalTrials.gov. Only 34% reported results in any format within 24 months of completion. Of 200 publications, 72% were freely accessible, and 23% of 100-article subsample included stakeholder engagement statements. Funder website sample yielded insufficient metadata for analysis.

Conclusion: Monitoring GHR is feasible using registries and journals, though funder websites provide limited tracking. While open access rates are encouraging, timely reporting and engagement documentation remain weak. These results highlight opportunities for developing GHR-specific monitoring approaches through collaborative efforts among global stakeholders.

Strengths and Limitations of this study

- We explored data from three distinct sources (clinical trial registries, global health journals, and global health funder websites), introducing a methodology suitable for assessing GHR studies using transparency and engagement indicators.
- Our findings provide empirical data while highlighting the need for expanded collaboration to refine indicators and methodologies, enhancing the contextual relevance of GHR monitoring.
- Trials from ClinicalTrials.gov were classified by location (LMIC vs. HIC), with two-thirds of selected trials from LMICs, ensuring equitable representation and addressing historical disparities in research quality assessments.
- We have provided detailed steps, accompanying code and data to support reproducibility.
- The study uses a sample of studies to demonstrate the feasibility of the methods, which can limit the generalizability of the findings.

For peer review only

For peer review only - http://bmjopen.bmj.com/site/about/guidelines.xhtml

1. Introduction

Global health research (GHR) is a collaborative, transnational research approach aimed at promoting health equity for all [1]. The field addresses major health challenges that affect large populations worldwide, often focusing on severe conditions with substantial impact on mortality and quality of life, particularly in resource-limited settings. Recent surges in GHR activities, especially spurred by the COVID-19 pandemic, have highlighted both the field's potential and its challenges in delivering timely evidence for decision-making.

Research transparency and stakeholder engagement are crucial elements across all fields of health research. In GHR, these practices take on additional dimensions due to the field's unique characteristics. For instance, many global health conditions affect substantial populations across different regions and cultural contexts, making rapid knowledge translation particularly important. Limited research infrastructure in many settings and historical power imbalances between researchers and communities further emphasize the need for transparent research practices and meaningful stakeholder engagement.

The timely dissemination of research findings through transparent practices such as prospective registration, comprehensive results reporting, and open-access publication is essential for evidence-based healthcare globally. In GHR, barriers to accessing research findings can be especially problematic—paywalled publications may be inaccessible to healthcare providers, researchers, and patients in low-resource settings, potentially widening rather than reducing health inequities. Similarly, the transnational and cross-cultural nature of GHR makes genuine stakeholder engagement particularly relevant for ensuring research relevance, cultural appropriateness, and eventual implementation.

Another defining characteristic of GHR is collaboration. The transnational and cross-sectoral nature of the field means that progress often depends on collective action across institutions, regions, and disciplines. Such collaboration has already proven effective in addressing major health challenges. Examples include the scale-up of antiretroviral therapy and cervical cancer screening, which originated in high-income countries (HIC) and were later adapted to resource-limited settings [2 3]. In return, innovations pioneered and scaled from low-and middle-income countries (LMIC) under resource-constrained conditions—including development of modern oral rehydration solution, evolution of kangaroo mother care—have been adopted globally as low-cost, effective interventions [4 5]. Given this central role of collaboration, robust monitoring is essential to ensure accountability, transparency, and meaningful engagement of stakeholders in order to maximize impact.

Historically, global health monitoring was primarily led by major inter-governmental agencies. However, academic research has increasingly complemented these efforts, enhancing the methods and rigour of global health measurement to support better decision-making by agencies, governments, and donors [6]. While significant efforts have been made to monitor trial transparency and engagement in Europe and North America [7-12], gaps remain in assessing these practices on a global scale [13]. For instance, the EU TrialsTrackers (http://eu.trialstracker.net/) specifically monitor whether summary results are posted on the EU clinical trials register and national-level dashboards have also been developed, providing a detailed breakdown of practices and helping stakeholders identify and address areas needing improvement [14 15]. Similar GHR monitors can help assess the current landscape, highlight actionable areas, and guide stakeholders—including patients, researchers, physicians, and global leaders—in making informed decisions.

Improved global health monitoring further requires new technologies and methods, as well as established norms and standards to facilitate global reporting [16]. To build an effective monitoring system for global health, it is important to first define what constitutes global health studies and assess them with appropriate indicators. We explored three methods for generating a sample of global health studies: (a) clinical trial registries focusing on global health conditions or diseases affecting populations globally, (b) publications from global health journals, and (c) research outputs mentioned on global health funders' websites. Using indicators established in previous studies [9-12], we assessed a sample of these studies to provide empirical data. We aim to demonstrate how past methodologies for monitoring clinical research can be applied to GHR, highlighting opportunities and challenges to enhance the implementation of monitoring systems in the GHR.

For peer review only - http://bmjopen.bmj.com/site/about/guidelines.xhtml

2. Methods

2.1. Sampling Strategy

We explored three complementary approaches to identify global health research studies: (a) clinical trial registries, using selected global health conditions as proxy, (b) publications from journals with an explicit global health focus, and (c) research outputs listed on global health funder websites. Each source offered distinct advantages and challenges for identifying and tracking global health studies. Below, we describe the methodology and purpose.

2.1.1 **Clinical trial registry with a disease-based approach**:

We systematically searched trial registry of ClinicalTrials.gov for interventional trials with start date between January 1, 2008 to completion date March 31, 2019. This eligibility requirement was based on the mandate for clinical trial registration in registries under both US laws and journal policies following ICMJE guidelines [17] passed on January 1, 2008. For our analysis, we focused on two health conditions known to have significant global burdens: maternal health issues (including postpartum depression, maternal sepsis, and maternal anaemia) and tuberculosis. Trials were identified using relevant MeSH terms for each condition (see supplement S1 for details). These conditions were chosen based on their significant global health burden and their relevance to our ongoing larger research project, where one of our team members has a specific interest in maternal health, particularly postpartum conditions. We excluded international multicentric trials, observational studies, and those with missing location or site information, as well as withdrawn studies. Trial locations were categorized as HIC or LMIC according to the World Bank country classification [18]. For our exploratory analysis, we randomly selected 100 trials per condition. To address the imbalance, with more than half of the trials originating from HICs, we ensured that two-thirds of the selected trials were from LMICs. This approach ensured equitable representation and also addressed the historical disparity in research quality assessments between HICs and LMICs. In this sample, we aim to demonstrate the feasibility of evaluating registration practices and the timeliness of result reporting by examining both summary results and publications from a sample of clinical trials within a global health context.

To locate trial publications, two independent reviewers (NH and SSY) followed a predefined search manual to identify the earliest publications of trial results (see supplement S2 for search manual). Searches were conducted in both the registry and Google, using trial IDs and relevant terms. Eligible publications included peer-reviewed articles or preprints with over 500 words, matched to the trial by design, population, intervention, and comparator (if applicable). Additionally, the publication's primary outcome measure (or first mentioned outcome) had to be listed as an outcome measure in the registration, whether classified as primary or not.

2.1.2 **Global health journals**:

Our second approach focused on generating a sample of global health studies from peer-reviewed journals specializing in global health. We identified 20 journals that either had 'global health' or 'international health' in their title (see supplement S3 for global health journals list). Using the Cochrane Highly Sensitive Search Strategy (Box 3b strategy), we located randomized trials published after January 1st, 2011, in PubMed and cross-referenced these results with our selected journals. Including trial publications post-2011 allowed a three-year window for dissemination of results, following the ICMJE mandate for prospective trial registration, effective from 2008. Our search yielded 1,125 records of trials published after 2011. An author (SSY) screened a random sample (n = 300), categorizing them as primary reports of trial results or secondary analyses by the original or different trial investigators. For analysis, we included the first 200 primary reports and secondary analyses by same investigators (see supplement S1 flowchart). This sample was used to evaluate how trial results are reported in GHR, examining adherence inclusion of data sharing, and community engagement statements.

2.1.3 **Global health funder website**:

The third method aimed to explore the use of global health funder websites as potential sources for generating global health studies. We investigated funder websites to determine if they provided sufficient metadata, such as trial registration numbers and registry links, to allow for the tracking of funded clinical trials and to assess transparency. This involved searching World RePORT [19], a database for tracking funding data for some largest biomedical funders, as well as a list of global health funders compiled by colleagues for a prior project. Our search revealed 20 global health funders, and 1 of them had explicit GH programs with available funding metadata, sufficient to track funded clinical trials (see supplement S4 for global health funders list).

For peer review only - http://bmjopen.bmj.com/site/about/guidelines.xhtml

We focused on the Fogarty International Center (FIC) at the National Institutes of Health (NIH), which supports global health research. Using their NIH RePORTER tool, we identified a list of 73 clinical trials and 9515 publications supported by 331 core projects. However, after applying the inclusion criterion with regard to date, the number of relevant trials was very limited, leading us to exclude this sample from our analysis (see Table 1 for overview).

Table 1: Established indicators used for assessing transparency and stakeholder engagement in global health research samples

**Indicator**	**Details**	**Assessment**	**Impact**
**Follow-up of clinical trials from clinical trial registry with a disease-based approach**			
Time to registration	Before any clinical trial is initiated its details must be registered in a publicly available, free-to-access, searchable clinical trial registry complying with WHO's internationally agreed standards[1].	Automated check by comparing the trial registration date with the trial start date. We report on<br>1) before the trial starts (prospective registration),<br>2) within 60 days after the trial starts (early registration),<br>3) after 60 days (late registration)	Transparency, unbiased reporting
Result reporting	Results can be reported in the following way: Published articles in a peer-reviewed journal (24 months from trial completion); Trial results uploaded in the trial registry (12 months from trial completion)[1].	Automated check for summary result reporting in the registry and manual search[2] for published articles. We report on<br>1) Summary result (SR) in registry<br>2) Journal publication<br>3) SR or journal publication within 1 year after trial completion<br>4) SR or journal publication within 2 years after trial completion<br>5) SR or journal publication within 4 years after trial completion	Transparency, informed decision making
Open access	Publications describing clinical trial results should be open-access[1].	Automated check for publication status (open access) for manually searched research publications of clinical trials.	Transparency, accessibility
**Analysis of clinical trial result publication in global health journals**			
Stakeholder/ Community Engagement	Involvement of patients and other relevant stakeholders (such as caregivers, healthcare providers, patient advocacy groups, policymakers, and the community) in any phase of the clinical study process.	Manual review[3] of publications for stakeholder involvement statements.	Practical relevance, inclusivity

For peer review only - http://bmjopen.bmj.com/site/about/guidelines.xhtml

Open Access	Publications describing clinical trial results should be open-access[1].	Automated check for publication status (open access).	Transparency, accessibility
Data sharing statement	Data sharing, archiving, depositions, and accessibility of data in journal articles, with or without request.	Automated check for declarations on data sharing and availability.	Transparency, reproducibility
Code sharing statement	Support sharing of code whenever appropriate.	Automated check for open code statements.	Transparency, reproducibility

[1] Text referenced from WHO Joint statement on public disclosure of results from clinical trials (https://www.who.int/news/item/18-05-2017-joint-statement-on-registration).
[2] Manual searches for locating result publications are done by two according to the manual (see supplement S2).
[3] Manual searches for screening studies with stakeholder engagement are done according to the manual (see supplement S5).

2.2. Software

We used the aactr R package [Version 0.0.0.9000] [20] to assess registration and summary result reporting in the registry of trials. The R packages oddpub [Version 7.2.2] [21] and roadoi [Version 0.7.3] [22] were used to assess availability statements and open access status, respectively, in trial publications. Map was created using the free and open source QGIS [Version 7.2.2] [23]. Data cleaning steps and statistical analyses were performed using R [Version 4.3.2] [24]. All analysis scripts are available under an open license on GitHub https://github.com/quest-bih/prodigy, and the underlying data are accessible via the Open Science Framework (OSF) at https://doi.org/10.17605/OSF.IO/HQDNS [25].

3. Results

This section provides an independent analysis of two samples: the first focuses on interventional trials from ClinicalTrials.gov, while the second examines trial result publications from global health journals. Data from the third funder website sample was limited and not analyzed.

3.1 Follow-up of clinical trials from clinical trial registry

We included data from 200 interventional trials (2009 – 2019) with 2/3rd representation from LMIC, enrolling 115,929 participants. Dominant trial characteristics were small sample size (<100) and open labelled (Table 2). The most common trial locations were the United States (n = 25), South Africa (n = 24), and China (n = 23) (Figure 1).

Table 2 Characteristics of included clinical trials

**Trial characteristic[1]**	**Tuberculosis (n =100)**	**Maternal Health Conditions (n = 100)**	**All trials, N= 200**
**Recruitment status**			
Completed	79 (79%)	76 (76%)	155 (77.5%)
Terminated	2 (2%)	8 (8%)	10 (5%)
Unknown status	19 (19%)	16 (16%)	35 (17.5%)
**Enrolment**			

For peer review only - http://bmjopen.bmj.com/site/about/guidelines.xhtml

1- 100	49 (49%)	40 (40%)	89 (44.5%)
100 - 500	32 (32%)	44 (44%)	76 (38%)
>500	19 (19%)	16 (16%)	35 (17.5%)
**Masking** (missing, n =1)			
Open label	62 (62%)	53 (53%)	115 (57.5%)
Single	3 (3%)	16 (16%)	19 (9.5%)
Double	8 (8%)	9 (9%)	17 (8.5%)
Triple	7 (7%)	5 (5%)	12 (6%)
Quadruple	19 (19%)	17 (17%)	36 (18%)
**Design** (missing, n =29)			
Non-randomized	10 (10%)	15 (15%)	25 (12.5%)
Randomized	76 (76%)	70 (70%)	146 (73%)
**Phase**			
I	19 (19%)	5 (5%)	24 (12%)
I-II	3 (3%)	1 (1%)	4 (2%)
II	21 (21%)	14 (14%)	35 (17.5%)
II-III	2 (2%)	2 (2%)	4 (2%)
III	9 (9%)	13 (13%)	22 (11%)
IV	11 (11%)	24 (24%)	35 (17.5%)
Not applicable	35 (35%)	41 (41%)	76 (38%)

[1]Total count may differ between categories because of missing data (number missing shown if >0).

For peer review only

For peer review only - http://bmjopen.bmj.com/site/about/guidelines.xhtml

Trial registration and result reporting

Of the 200 trials, 74 (37%) were registered prospectively. Regarding result reporting, from all 200 trials that were completed until 2019 or earlier and thus had 4 or more years to report results, 129 trials (65%) had results published in journals, and 30 (15%) reported summary results in registries. The number of trials that reported any results (either via journals or summary results) within one, two, or four years after completion was 29 trials (15%), 67 trials (34%) and 116 (58%) (Table 3).

For journal result publications identified using a search manual, the reviewer independently located relevant publications with an interrater reliability of 83%. When different publications were selected, 100% agreement was reached on selecting the earliest matching publication. All matched publications were peer-reviewed articles, with majority specifically, 60% (78 /129), linked in the registry.

Table 3 Registration and result reporting of included clinical trials

Indicators	Result (n, %)
	All trials, N= 200
**Time to registration**	
Prospective registration	74 (37%)
Within 60 days after trial start	50 (25%)
>60 days after trial start	76 (38%)
**Result reporting**	
Journal Publication	129 (65%)
Summary result (SR) in registry	30 (15%)
Result reporting within (SR or Journal) 1 year after completion	29 (14.5%)
Results reporting (SR or Journal) within 2 years after completion	67 (34%)
Result reporting (SR or Journal) within 4 years after completion	116 (58%)
Open Access (n = 125[1])<br>[2]Gold:22, Green: 20, Hybrid: 13, Bronze:10, Closed: 15, NA: 45	65 (52%)

[1] For all 129 journal publications, we could find a DOI for 125 articles
[2] Gold: articles published in fully open-access journals; Green: articles self-archived in repositories or institutional archives; Hybrid: articles published in subscription journals with an open-access option; Closed: articles not publicly accessible.

3.2 Analysis of trial results in publications from GH journals

Of the 200 trial publications in global health journals between 2011 to 2023, 72% (144) were Gold Open Access (freely accessible), 22.5% (45) were Hybrid Open Access (partially accessible), and 1.5% (3) were closed access. We were able to download 127 of the 200 PDFs to assess data and code availability. Among these, 59% (75) included a data-sharing statement, often specifying availability upon request, and only 0.8% (1) included a code-sharing statement. A random sample of 100 articles was reviewed for stakeholder engagement statements using a predefined manual(see supplement S5 search manual), with 23% (23) reporting relevant content (Table 4).

Table 4: Transparency and community engagement in included trial result publication

Indicators	Result (n, %)
Open Access (n = 200)<br>[1]Gold: 144, Green: 8, Hybrid:45, Closed:3.	197 (99%)
Data sharing statement including 'available upon request' (n = 127[2])	75 (59%)
Code sharing statement (n = 127[2])	1 (0.8%)
Stakeholder engagement statement (n = 100[3])	23 (23%)

1Gold: articles published in fully open-access journals; Green: articles self-archived in repositories or institutional archives; Hybrid: articles published in subscription journals with an open-access option; Closed: articles not publicly accessible.
[2]For all 200, we had 127 PDFs.
[3]Checked for a random sample of 100 publications

For peer review only - http://bmjopen.bmj.com/site/about/guidelines.xhtml

4. Discussion

This study aimed to explore approaches for monitoring transparency and stakeholder engagement in GHR while providing empirical data on the current state of these practices. We tested three different sampling strategies and found that trial registries focusing on global health conditions and global health journals provide viable data sources for monitoring, while funder websites currently lack sufficient metadata for systematic tracking.

Among 200 registered trials identified through ClinicalTrials.gov focusing on selected global health conditions, 37% were prospectively registered, and 65% had published results in journals, though only 15% reported summary results. Within 24 months of trial completion, only 34% reported their result in any format and thus demonstrated timely results reporting. These findings on timely results reporting are comparable to previous studies examining clinical trial transparency in other contexts, such as German university medical centers (43%) [10], Nordic countries (52%) [9], and US academic medical centers (36%) [7]. Among identified publications, 60% were linked to their registry entries, indicating moderate but improvable compliance with WHO recommendations for result traceability [26]. While the overall rates are similar to other contexts, timely results reporting is particularly important in GHR, given that many global health conditions represent a high global burden of disease (GBD). Although it would be problematic to claim that timely reporting is more important in some research fields than others, the often substantial disease burden addressed in GHR emphasizes the need for rapid knowledge translation to inform evidence-based healthcare decisions.

For the sample of trial result publications from selected global health journals, the observed high rate of open-access publications (99%) is particularly encouraging, as paywalled research results would disproportionally affect healthcare providers and researchers in low-resource settings. This rate markedly exceeds the average of 28% open access found across scholarly literature [27]. While this high rate may partly reflect our sampling of global health journals with established open-access policies and increasing pressure on global health researchers to publish transparently, it nevertheless demonstrates substantial progress in making GHR findings widely accessible. The increasing adoption of institutional monitoring tools, as demonstrated by Franzen et al. [14], could help track and improve transparency in GHR. Such tools should incorporate core open science practices recently identified through expert consensus while considering GHR-specific needs [28].

The baseline measurement of stakeholder engagement reporting from the global health journal sample— 23% of the 100 publications assessed—demonstrates that evaluating GHR using this indicator is feasible and also provides important insights for future development. Given that GHR often involves transnational and cross-cultural research contexts, meaningful engagement of local communities and stakeholders is particularly crucial. Encouragingly, recent years have seen a positive push toward advancing patient and stakeholder engagement (PSE) in GHR. For example, initiatives such as the RESPIRE (Global Health Research Unit on Respiratory Health) group have developed structured protocols and practical tools, including templates for designing, implementing, and evaluating stakeholder engagement plans that are tailored to local contexts and linked to planned outcomes. Such resources help translate field experiences into actionable guidance [29]. While our analysis establishes a baseline for PSE reporting in GHR, more detailed analyses, such as those demonstrated by Weschke et al. [30], are needed to assess the quality and impact of these practices, providing valuable insights that can inform future improvements.

This study had several strengths and limitations. First, the disease-based sampling strategy using trial registries proved effective for identifying GHR studies but required careful selection of MeSH terms representing the conditions. A key methodological challenge was ensuring balanced representation between high- and low-income countries. Our approach of oversampling LMIC-based studies (2/3 of the trial registry sample) provides one potential solution, though optimal sampling strategies for GHR monitoring should be further discussed with stakeholders. Second, the journal-based approach successfully identified GHR publications but is limited by the need to rely on "global health journals" to represent global health research. For screening of open data and open code, we were able to automatically download 127 out of 200 PDFs. These were then screened using the ODDPub tool, which has a reported sensitivity of 73% [21]. Manual validation was not performed - neither for downloading the remaining PDFs nor for identifying potential false negatives. This decision was consistent with our aim to demonstrate the feasibility of these methods in GHR monitoring in a scalable and often preferable

For peer review only - http://bmjopen.bmj.com/site/about/guidelines.xhtml

automated manner, rather than to provide validated quantitative estimates. Finally, the funder-based approach highlighted gaps in tracking systems that limit comprehensive monitoring. While some global health funder websites provide lists of funded studies, they often lack essential metadata, such as trial registration numbers or the registry in which the trial is registered, which currently prevents automated assessment of transparency practices for these studies.

5. Conclusion

In conclusion, our study revealed that monitoring GHR is feasible using trial registries and journals, although funder websites provide limited tracking. While open access rates are encouraging, timely reporting and engagement documentation remain weak. We assessed only a sample of studies to provide empirical data on how GHR can be monitored, demonstrating the feasibility of these methods. However, global health research is inherently collaborative, requiring active input from researchers and stakeholders. Future efforts should identify additional indicators for monitoring, address challenges in informing researchers about their performance in transparency and expand on our findings using new samples and methodologies. Continued monitoring can drive improved practices, fostering more transparent and inclusive GHR to support evidence-based global health advancements.

6. Data sharing

All the code for this study is available via open-source licensing on GitHub (https://github.com/quest-bih/prodigy). Data generated in this study are openly available under the Creative Commons Attribution 4.0 International license in OSF (https://doi.org/10.17605/OSF.IO/HQDNS).

7. Declaration of interests

We declare no competing interests.

8. Patient and public involvement

Patients and/or the public were not involved in the design, or conduct, or reporting, or dissemination plans of this research.

9. Patient consent for publication

Not applicable.

10. Ethics approval

Not applicable.

11. Acknowledgments

This study is part of the PRODIGY project and we would like to thank Malek Bajbouj, Isabel Dziobek, Eric Hahn, Thi Minh Tam Ta, María Jose Lobeda-Garzón, as well as Sarah Weschke, Vladislav Nachev and Delwen Franzen, who contributed their ideas and knowledge to make this study possible.

12. Author contributions

SSY — Conceptualization, Investigation, Methodology, Formal Analysis, Writing (Original Draft), Writing (Review & Editing). NH — Investigation, Writing (Review & Editing). DS — Conceptualization, Methodology, Writing (Review & Editing), Supervision, Funding Acquisition. Guarantor for the overall content: SSY.

13. Funding

For peer review only - http://bmjopen.bmj.com/site/about/guidelines.xhtml

This work was funded by the Berlin University Alliance (BUA). DS received funding for a work package within the PRODIGY project. The funder had no role in the study design, data collection and analysis, decision to publish, or preparation of the manuscript.

14. References

1. Koplan JP, Bond TC, Merson MH, et al. Towards a common definition of global health. *Lancet* 2009;373(9679):1993-5. doi: 10.1016/S0140-6736(09)60332-9 [published Online First: 2009/06/06]
2. El-Sadr WM, Holmes CB, Mugyenyi P, et al. Scale-up of HIV treatment through PEPFAR: a historic public health achievement. *J Acquir Immune Defic Syndr* 2012;60 Suppl 3(Suppl 3):S96-104. doi: 10.1097/QAI.0b013e31825eb27b [published Online First: 2012/09/14]
3. Puricelli Perin DM, Vogel AL, Taplin SH. Assessing Knowledge Sharing in Cancer Screening Among High-, Middle-, and Low-Income Countries: Insights From the International Cancer Screening Network. *J Glob Oncol* 2019;5:1-8. doi: 10.1200/JGO.19.00202 [published Online First: 2019/10/05]
4. Bhattacharya SK. History of development of oral rehydration therapy. *Indian J Public Health* 1994;38(2):39-43. [published Online First: 1994/04/01]
5. Kostandy RR, Ludington-Hoe SM. The evolution of the science of kangaroo (mother) care (skin-to-skin contact). *Birth Defects Res* 2019;111(15):1032-43. doi: 10.1002/bdr2.1565 [published Online First: 2019/08/17]
6. Horton R, Murray C, Frenk J. A new initiative and invitation for health monitoring, tracking, and evaluation. *Lancet* 2008;371(9619):1139-40. doi: 10.1016/S0140-6736(08)60498-5 [published Online First: 2008/04/09]
7. Chen R, Desai NR, Ross JS, et al. Publication and reporting of clinical trial results: cross sectional analysis across academic medical centers. *BMJ* 2016;352:i637. doi: 10.1136/bmj.i637 [published Online First: 2016/02/19]
8. Macleod MR, Michie S, Roberts I, et al. Biomedical research: increasing value, reducing waste. *Lancet* 2014;383(9912):101-4. doi: 10.1016/S0140-6736(13)62329-6 [published Online First: 2014/01/15]
9. Nilsonne G, Wieschowski S, DeVito NJ, et al. Results reporting for clinical trials led by medical universities and university hospitals in the nordic countries was often missing or delayed. *J Clin Epidemiol* 2025;181:111710. doi: 10.1016/j.jclinepi.2025.111710 [published Online First: 2025/02/04]
10. Riedel N, Wieschowski S, Bruckner T, et al. Results dissemination from completed clinical trials conducted at German university medical centers remained delayed and incomplete. The 2014 -2017 cohort. *J Clin Epidemiol* 2022;144:1-7. doi: 10.1016/j.jclinepi.2021.12.012 [published Online First: 2021/12/16]
11. Strzebonska K, Wasylewski MT, Zaborowska L, et al. Results dissemination of registered clinical trials across Polish academic institutions: a cross-sectional analysis. *BMJ Open* 2020;10(1):e034666. doi: 10.1136/bmjopen-2019-034666 [published Online First: 2020/01/25]
12. Wieschowski S, Riedel N, Wollmann K, et al. Result dissemination from clinical trials conducted at German university medical centers was delayed and incomplete. *J Clin Epidemiol* 2019;115:37-45. doi: 10.1016/j.jclinepi.2019.06.002 [published Online First: 2019/06/14]
13. Tijdink JK, Horbach S, Nuijten MB, et al. Towards a Research Agenda for Promoting Responsible Research Practices. *J Empir Res Hum Res Ethics* 2021;16(4):450-60. doi: 10.1177/15562646211018916 [published Online First: 2021/05/27]
14. Franzen DL, Carlisle BG, Salholz-Hillel M, et al. Institutional dashboards on clinical trial transparency for University Medical Centers: A case study. *PLOS Medicine* 2023;20(3):e1004175. doi: 10.1371/journal.pmed.1004175

For peer review only - http://bmjopen.bmj.com/site/about/guidelines.xhtml

15. Jeangirard E. Monitoring Open Access at a national level: French case study. ELPUB 2019 23rd edition of the International Conference on Electronic Publishing. Marseille, France: African Society in Digital Science, 2019.
16. Murray CJ, Lopez AD, Wibulpolprasert S. Monitoring global health: time for new solutions. *BMJ* 2004;329(7474):1096-100. doi: 10.1136/bmj.329.7474.1096 [published Online First: 2004/11/06]
17. International Committee of Medical Journal Editors (ICMJE). Clinical trials registration [Available from: https://www.icmje.org/about-icmje/faqs/clinical-trials-registration/ accessed 23rd May 2024].
18. World Bank. World Bank Country and Lending Groups [Available from: https://datahelpdesk.worldbank.org/knowledgebase/articles/906519-world-bank-country-and-lending-groups accessed 7th July 2024].
19. Adam T, Akuffo H, Carter JG, et al. World RePORT: a database for mapping biomedical research funding. *Lancet Glob Health* 2020;8(1):e27-e29. doi: 10.1016/S2214-109X(19)30459-0 [published Online First: 2019/12/17]
20. Salholz-Hillel M. aactr R package. Version 0.0.0.9000. GitHub [Available from: https://github.com/maia-sh/aactr accessed 2nd May 2023].
21. Riedel N, Kip, M. and Bobrov, E. ODDPub – a Text-Mining Algorithm to Detect Data Sharing in Biomedical Publications. *Data Science Journal* 2020 doi: 10.5334/dsj-2020-042
22. Jahn N. roadoi: Find Free Versions of Scholarly Publications via Unpaywall. Version 0.7.3. [Available from: https://docs.ropensci.org/roadoi/ accessed 2nd May 2023].
23. QGIS Development Team. QGIS, Free and Open Source Software. Version 7.2.2. [Available from: https://www.qgis.org].
24. R Core Team. R: A language and environment for statistical computing. Version 4.3.2. [Available from: https://www.R-project.org].
[dataset] 25. Yerunkar S, Strech D, Hildebrand N. Dataset from: Towards monitoring of global health research: an exploratory analysis of transparency and stakeholder engagement. OSF; 2025 Nov 4. doi:10.17605/OSF.IO/HQDNS
26. World Health Organization. World Health Organization statement on public disclosure of results from clinical trials [Available from: https://www.who.int/news/item/18-05-2017-joint-statement-on-registration accessed 15 June 2024].
27. Piwowar H, Priem J, Lariviere V, et al. The state of OA: a large-scale analysis of the prevalence and impact of Open Access articles. *PeerJ* 2018;6:e4375. doi: 10.7717/peerj.4375 [published Online First: 2018/02/20]
28. Cobey KD, Haustein S, Brehaut J, et al. Community consensus on core open science practices to monitor in biomedicine. *PLoS Biol* 2023;21(1):e3001949. doi: 10.1371/journal.pbio.3001949 [published Online First: 2023/01/25]
29. Jackson T, Fernandes G, Williams S, et al. Stakeholder engagement champions: a locally-driven model for building impactful and sustainable relationships in global health research. *J Glob Health* 2025;15:03026. doi: 10.7189/jogh.15.03026 [published Online First: 2025/06/06]
30. Weschke S, Franzen DL, Sierawska AK, et al. Reporting of patient involvement: a mixed-methods analysis of current practice in health research publications using a targeted search strategy. *BMJ Open* 2023;13(1):e064170. doi: 10.1136/bmjopen-2022-064170 [published Online First: 2023/01/21]

15. Figure legends

Figure 1 Geographic distribution of included clinical trials.

For peer review only - http://bmjopen.bmj.com/site/about/guidelines.xhtml

For peer review only

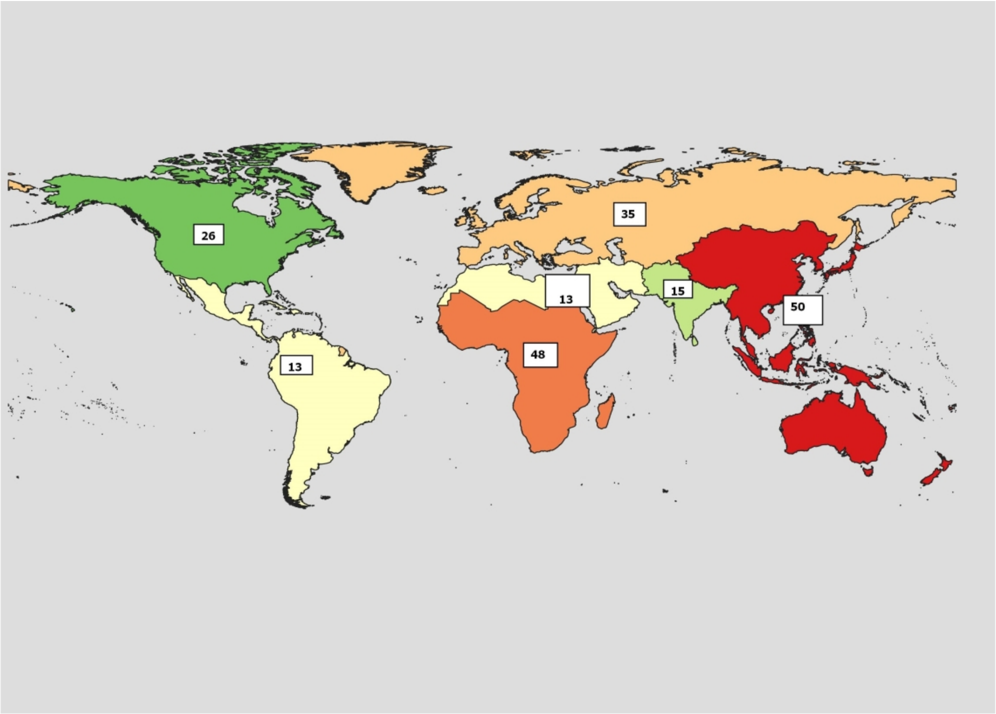

Figure 1 Geographic distribution of included clinical trials.

256x183mm (300 x 300 DPI)

Supplementary file

Supplement S1: Search strategies to identify global health studies

1. **Clinical trial registry with a disease-based approach**

On 13th April 2024, using the ClinicalTrials.gov web front (https://clinicaltrials.gov/), we searched studies by Condition/disease (MeSH terms) in the following way:

Condition	N
Postpartum Depression (also searched for **Depressive Disorders**, **Puerperal**, and **Post Natal Depression**. See Search Details)	450
Maternal Sepsis (also searched for **Depressive Disorders**, **Puerperal**, and **Post Natal Depression**. See Search Details)	1403
Maternal Anemia (also searched for **Anemia of pregnancy**, **Pregnant**, **Anemia during pregnancy** and more. See Search Details)	437
Tuberculosis (See Search Details)	1391

2. **Global Health Journal**

On 13th April 2024, using the Cochrane Highly Sensitive Search Strategy (Box 3b strategy), we identified randomized trials in PubMed and cross-referenced these results with articles from 20 global health journals.

Box 3. b Cochrane Highly Sensitive Search Strategy for identifying randomized trials in MEDLINE: sensitivity- and precision-maximizing version (2008 revision); PubMed format

#1 randomized controlled trial [pt]

#2 controlled clinical trial [pt]

#3 randomized [tiab]

#4 placebo [tiab]

#5 clinical trials as topic [mesh:noexp]

#6 randomly [tiab]

#7 trial [ti]

#8 #1 OR #2 OR #3 OR #4 OR #5 OR #6 OR #7

#9 animals [mh] NOT humans [mh]

#10 #8 NOT #9

PubMed search syntax:
[pt] denotes a Publication Type term;
[tiab] denotes a word in the title or abstract;
[sh] denotes a subheading;
[mh] denotes a Medical Subject Heading (MeSH) term ‘exploded’;
[mesh:noexp] denotes a Medical Subject Heading (MeSH) term not ‘exploded’;
[ti] denotes a word in the title.

For peer review only

Supplementary file

PubMed terms used to extract articles

((((("The Lancet. Global health"[Journal]) OR ("Journal of global health"[Journal])) OR ("Globalization and health"[Journal])) OR ("Annals of global health"[Journal])) OR ("pathogens and global health"[Journal])) OR ("Global public health"[Journal])) OR ("Global health action"[Journal])) OR ("Global health promotion"[Journal])) OR ("BMJ global health"[Journal])) OR ("Global pediatric health"[Journal])) OR ("Global health, epidemiology and genomics"[Journal])) OR ("Global health research and policy"[Journal])) OR ("The Central African journal of medicine"[Journal])) OR ("Clinical epidemiology and global health"[Journal])) OR ("Global journal of health science"[Journal])) OR ("Global mental health (Cambridge, England)"[Journal])) OR ("international journal of travel medicine and global health"[Journal])) OR ("plos global public health"[Journal])) OR ("Global journal of health science"[Journal])) OR ("journal of global health perspectives"[Journal])) OR ("Global health journal (Amsterdam, Netherlands)"[Journal])) and (((randomized controlled trial[Publication Type]) OR (controlled clinical trial[Publication Type]) OR (randomized[Title/Abstract]) OR (placebo[Title/Abstract]) OR (clinical trials as topic[mesh:noexp]) OR (randomly[Title/Abstract]) OR (randomly[Title/Abstract]) OR (trial[Title])) NOT (animals [mh] NOT humans [mh]))) NOT review [pt]) AND ((english[Filter]) AND (2011:2024[pdat]))) Filters: English, from 2011 – 2024

Note: We additionally excluded articles (n = 9) from “The Central African journal of medicine” journal, which was not explicitly a global health journal. Please see “13-4-2024-global-health-article” for details.

For peer review only

Supplementary file

Supplement S1 Global health journal analysis flowchart

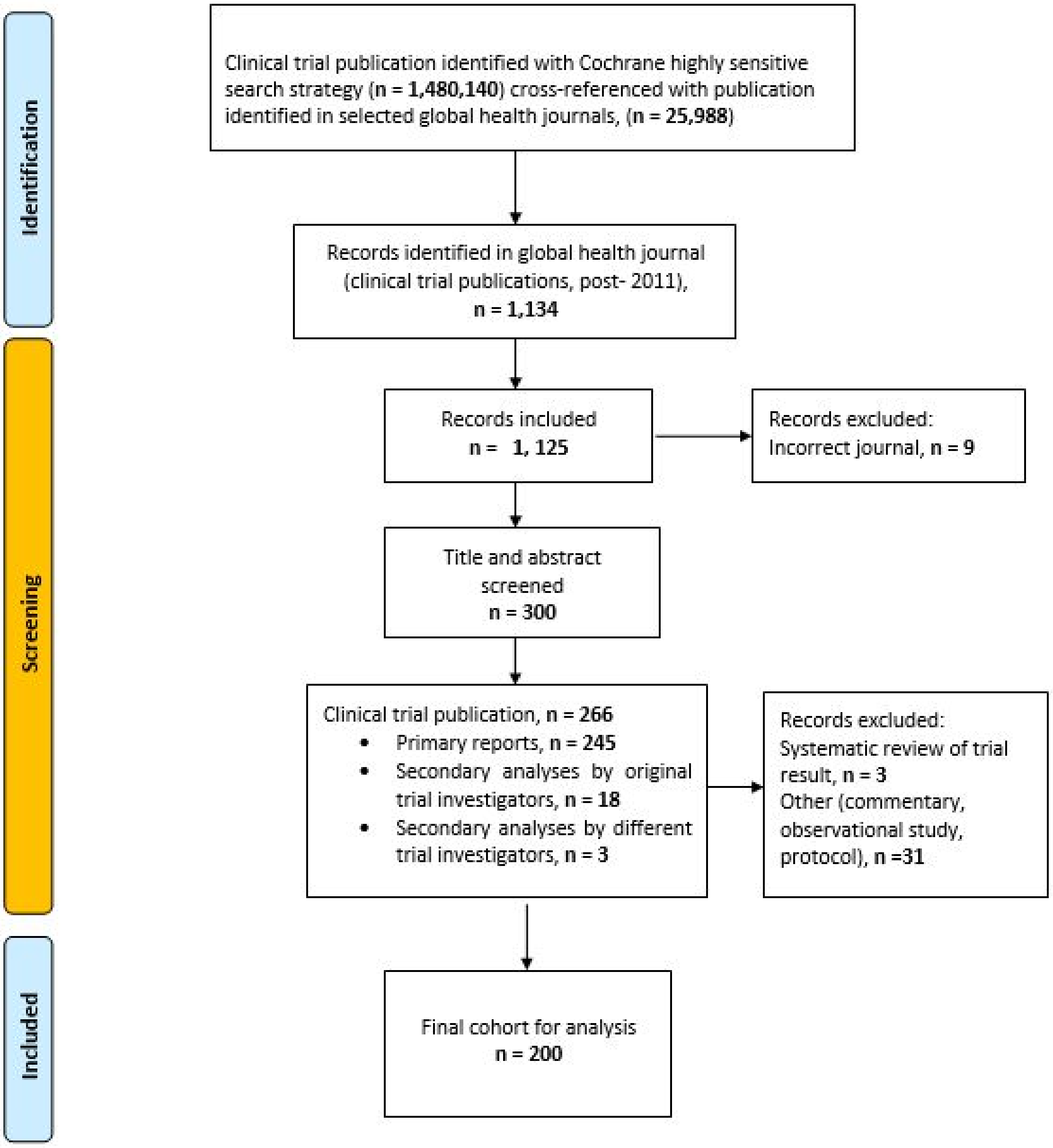

Supplementary file

3. Global health funder website

On 13th April 2024, using the NIH RePORTER tool's 'Advanced Project Search' (https://reporter.nih.gov/advanced-search) and selecting the Funding tab, with Agency/Institute/Center set to "John E. Fogarty International Center for Advanced Study in the Health Sciences (FIC)," the search results included:

- 73 clinical trials,
- 9,515 publications,
- Supported by 331 core projects.

For peer review only

Supplement S2: Search manual for clinical trial result publication

Our goal is to identify result publications for clinical trials using the criteria mentioned in this manual. We will strive to find the earliest result publication that matches the trial in a reasonably unambiguous way and reports its results.

Identification steps

Identification of publications linked to each clinical trial will be done in a 3-step process. Go through steps 1-2 for all trials. Continue with step 3 only if no eligible results publication has been found during steps 1-2.

Check the ClinicalTrials.gov website under the tab 'Publications' to identify results publications linked in the registration. At ClinicalTrials.gov, these are either actively submitted by the sponsor/investigator or automatically indexed. Links may lead to reviews or other background literature, which will not be counted as results publications.

2) Google search using the main NCT ID. The first page (or a minimum of 10 first hits) will be screened.

3) Google search using two combinations of terms from the registry, e.g., title, principal investigator name, intervention/treatment, disease/symptoms, or other. Use combinations that you believe are specific enough to yield a valid match. The first page will be screened for each combination.

At each step, search for eligible results publication, except those with the same URL or same DOI as one previously extracted for the same trial. Among unique eligible result publications found at each step, enter only one with the earliest publication date.

Earliest Publication date**?:** The earliest date an article was published. Make sure you are checking to see if there is an "Epub" or "Online First" date (sometimes you need to check both PubMed and the Journal website) because, for a lot of journals, there will be both the date it was first published online and then a subsequent date it was published in print (for journals that still do print issues!) We want the earliest it shows up in any format.

Supplementary file

For all steps, if a publication is found it will be verified that it is indeed a results publication for the study and not only a mention of the NCT ID in a different context (e.g. review listingto th ongoing or completed trials). This verification is performed based on the title and abstract of the publication and if needed by referring to the full text. If the publication is not a results publication, the publication search will continue. If no hit occurs, we will proceed to the next step. Only if all three searches stay without results, the study will be characterized as "no publication found".

Extractions from publications

In case we identify a publication, we will extract manually the following identifiers: PubMed ID, DOI, URL and the earliest publication date. If only month and year are given, we will impute the day as the first day of the month.

Interrater reliability

Two reviewers (NH and SSY) will initially analyze the same 10 trials and the results will then be discussed to synchronize search strategy and reduce ambiguities. Both reviewers will independently extract data from all trials, and we will reach conclusions in the following manner:

1. **Same Publication Found by Both Reviewers with Different Earliest Dates:**
    - The publication with the earliest date will be counted.
2. **No Publication Found:**
    - If neither reviewer finds a publication, no publication will be recorded.
3. **Different Publications Found by Each Reviewer:**
    - Both reviewers must agree on the correct publication.
    - In cases where no agreement is reached, a third person will be consulted to decide on the correct publication.

Eligibility criteria for result publications

We will include results publications that correspond to both the following criteria

1. Is a peer-reviewed scientific publication or preprint with >500 words. Doctoral theses, congress abstracts, or other summaries are not counted as results publications unless fulfilling these criteria. Reviews not reporting primary data are not included.

2. Is matched to an eligible clinical trial on all criteria of

a.Study design (intervention model, randomization, masking)

b.Indication/population

c.Intervention/treatment

d.Comparator (if applicable)

For peer review only

Supplementary file

e.The publication's primary outcome measure needs to be listed as an outcome measure in the registration (whether as primary or not). If the publication has several primary outcome measures, the rule applies to at least one of them. If the publication has no primary outcome measure assigned, the rule applies to the first outcome measure mentioned.

Publications will be counted regardless of whether actual enrollment matches planned enrollment since this is often subject to change. Publications that report preliminary analyses for at least one predefined outcome will be counted. Also, publications reporting interim results or fewer study arms are counted (given that the overall study aim is still the same). Publications with reports of several trials combined (without disaggregating between trials) are not included.

For peer review only

Supplementary file

Supplement S3 Global health journals list

Sr no	Journal Title	Journal Description with focus on global health research	Include in study?
1	The Lancet Global Health	Our focus is on disadvantaged populations, be they whole economic regions or marginalised groups within otherwise prosperous nations, with a preference for the following topics: reproductive, maternal, neonatal, child, and adolescent health; infectious diseases, including neglected tropical diseases; non-communicable diseases; mental health; the global health workforce; health systems; surgery; and health policy.	Yes
2	Journal of Global Health	Aims to serve the community of researchers, funding agencies, international organizations, policy-makers and other stakeholders in the field of international health.	Yes
3	Globalization and Health	Provides a platform for research, knowledge sharing and debate on the topic of globalization and its effects on health, both positive and negative.	Yes
4	Annals of Global Health	Aims to advance and disseminate knowledge of global health, promote research and foster the prevention and treatment of disease worldwide.	Yes
5	Pathogens and Global Health	Deals with tropical diseases and medical and veterinary parasitology in their broadest aspects.	Yes
6	Global Public Health	The journal is broad-based and wide-ranging, including work that draws on the environmental health sciences; epidemiology; health policy and management; and the social sciences as applied to public health and medicine.	Yes
7	Global Health Action	Covers the research on public global health: Health information, health determinants, health interventions, environmental change and health, health systems and gender.	Yes

For peer review only

Supplementary file

8	Global Health Promotion	Presents theoretical research and practical papers on experiences, opinions and research leading to applications for health promotion and health education internationally.	Yes
9	BMJ Global Health	Covers all aspects of global health with particular interest in submissions that address the underfunded area of non-communicable diseases.	Yes
10	Global Pediatric Health	Focuses on health issues of children that are common to all regions of the world.	Yes
11	Global Health, Epidemiology, and Genomics	Dedicated to publishing and disseminating research that addresses and increases understanding of global and population health issues through the application of population science, genomics and applied technologies.	Yes
12	Global Health Research and Policy	Aims to rapidly disseminate high quality research to improve regional and global health.	Yes
13	Central Asian Journal of Global Health*	The journal provides a forum for discussion of all aspects of public health, medicine, and global health in Central Asia and around the world.	Yes (excluded later)
14	Clinical Epidemiology and Global Health	Aims to promote clinical epidemiology and global health, with particular emphasis on Indian and Asian countries.	Yes
15	Global Health Journal	Covers all aspects of global health	Yes
16	Global Journal of Health Science	Covers health science, health policy and services, preclinical medicine, clinical medicine, oral medicine, public health and preventive medicine, pharmacy, military and special medicine, medical and related biological research, education, and medical awareness in the public.	Yes
17	Global Mental Health	Aims to cover a broad application of 'the global point of view' of mental health issues.	Yes

For peer review only

Supplementary file

18	International Journal of Travel Medicine and Global Health	Publishes papers in all fields of travel medicine & global health aiming to increase the understanding, diagnosis and treatment of various disorders which can be presented, deteriorated, and healed in a travel. Aims to facilitate the exchange of ideas, hypothesis, techniques and information among all physicians.	Yes
19	PLOS Global Public Health	Publishes diverse research addressing global public health challenges and inequities	Yes
20	Global journal of health science	provide a platform for the global research community to share their findings, insights and views about all aspects of health science	Yes
21	Journal of global health perspectives	Journal of Global Health (JoGH) is a peer-review general medical journal focusing on issues relevant for global health.	Yes

*excluded later as rephrased to "The Central African Journal of Medicine" which is not global health journal per se

Supplementary file

Supplement S4: Global health funders list

**Funder name**	**Description in relation to global health research**	**Online resource to track grants (Yes/No)**	**Clinical trial funder**	**Trial registry number to track funded clinical trial (Yes/No)**	**Link to track grants or grants tracking system**	**Source**
Bill & Melinda Gates Foundation (BMGF)	We provide funding to organizations to achieve measurable impact in the fight against poverty, disease, and inequity around the world.	Yes	Yes	No	https://www.gatesfoundation.org/about/committed-grants	*U.S National Institutes of Health World RePORT
Canadian Institutes of Health Research (CIHR)	As part of its mission, CIHR seeks to foster effective Canadian involvement in international health research that benefits Canadians and the global community.	Yes	Yes	No	https://webapps.cihr-irsc.gc.ca/decisions/p/main.html?lang=en#fq={!tag=programtype2}programtype2%3A%22Randomized%20Controlled%20Trials%22&sort=namesort%20asc&start=0&rows=20	U.S National Institutes of Health World RePORT
European Commission (EC)	The European Commission manages the European Union research and innovation budget and supports research projects that span several years. The european union horizon	Yes	Yes	Yes	https://cordis.europa.eu/search/en	U.S National Institutes of Health World RePORT

For peer review only

Supplementary file

	funding focuses on tackling global challenges					
European & Developing Countries Clinical Trials Partnership (EDCTP)	The EDCTP2 programme supports clinical research, research capacity development and international networking in order to accelerate the clinical development of effective, safe, accessible, suitable and affordable medical interventions for poverty-related infectious diseases affecting sub-Saharan Africa.	Yes	Yes	Yes	https://www.edctp.org/edctp2-project-portal/	U.S National Institutes of Health World RePORT

For peer review only

Supplementary file

German Federal Ministry of Education and Research (BMBF)	The BMBF supports international research activities in sub-Saharan Africa and thus makes a targeted contribution to significantly improving medical care. The focus is on neglected and poverty-related diseases, among other things.<br><br>The fight against the increasing resistance of bacteria and other pathogens to medicines is also a key concern of the G7 countries. This is because widespread antibiotic resistance in particular poses a huge threat to global health. The G7 partners have therefore once again placed the fight against this resistance in the spotlight under the German presidency in 2022. Equally important is joint preparation for	Yes	Yes	No	https://www.gesundheitsforschung-bmbf.de/de/klinische-studien-3384.php	U.S National Institutes of Health World RePORT

For peer review only

Supplementary file

	epidemics or pandemics, which can be triggered by newly emerging pathogens that often jump from the animal kingdom to humans.					
Global Alliance for Chronic Diseases (GACD)	GACD is a global alliance of health research funders representing 80% of public health research funding worldwide	Yes	Yes	No	https://www.gacd.org/research/projects	U.S National Institutes of Health World RePORT
Japan Agency for Medical Research and Development (AMED)	The purpose of this organization is to carry out research and development in the medical field and to improve the environment for it, as well as to provide support, etc., in order to comprehensively and effectively develop the medical field.	Yes	Yes	TBD	https://amedfind.amed.go.jp/amed/index.html	U.S National Institutes of Health World RePORT

For peer review only

Supplementary file

Korea Health Industry Development Institute (KHIDI)	KHIDI supports initiatives to protect the rights and interests of foreign patients, and promotes the convenience of using medical care in Korea so that patients can receive safe and high-quality medical and healthcare services.	No	No	No	https://worldreport.nih.gov/wrapp/#/search?searchId=65f9be981a171a6207c251c3	U.S National Institutes of Health World RePORT
Medical Research Council (MRC)	Launched in April 2018, UK Research and Innovation (UKRI) is a non-departmental public body sponsored by the Department for Science, Innovation and Technology (DSIT). We aim to support UK and low and middle-income country scientists to establish partnerships, participate in global health research opportunities and respond to global challenges in biomedical and health research.	Yes	Yes	No	https://gtr.ukri.org/, https://www.ukri.org/what-we-do/browse-our-areas-of-investment-and-support/?category=mrc&closure_status=open	U.S National Institutes of Health World RePORT

For peer review only

Supplementary file

National Health Medical Research Council (NHMRC)	NHMRC is the key driver of health and medical research in Australia.We engage strategically with international funding agencies and participate in funding schemes that align with the NHMRC International Engagement Strategy 2023-2026.	Yes	Yes	No	https://www.nhmrc.gov.au/funding/data-research	U.S National Institutes of Health World RePORT
National Institutes of Health (NIH)	The Fogarty International Center at the U.S. National Institutes of Health (NIH) is dedicated to advancing the NIH mission by supporting and facilitating global health research conducted by U.S. and international investigators, building partnerships between health research institutions in the U.S. and abroad	Yes	Yes	Yes	https://reporter.nih.gov/search/N2a3NpquYEOspjX9UMAQdg/projects	U.S National Institutes of Health World RePORT
Swedish Research Council (SRC)	We fund research and research infrastructure in all scientific disciplines. We are also advisors to the	Yes	Yes	No	Swecris - Vetenskapsrådet (vr.se)	U.S National Institutes of Health World RePORT

For peer review only

Supplementary file

	Government on research policy issues and work to increase understanding of the long-term societal benefits of research.					
Wellcome Trust	Wellcome is a global charitable foundation established in 1936. That's why we're committed to funding ambitious global research projects that will transform our understanding of life, health, and wellbeing.	Yes	Yes	No	https://wellcome.org/grant-funding/funded-people-and-projects	U.S National Institutes of Health World RePORT
German Research Foundation (DFG)	Collaborative research that involves a diverse range of people from different fields of interest is key to progress in health science – and to achieving our aim of fostering a healthier, happier, world.	Yes	Yes	Yes	https://gepris.dfg.de/gepris/OCTOPUS	Global health funder list by collegeau
German Alliance for Global Health Research	GLOHRA aims to strengthen global health research in Germany by connecting a community of qualified	Yes	Yes	No	https://globalhealth.de/funded-projects.html	Global health funder list by collegeau

For peer review only

Supplementary file

	researchers, funding groundbreaking research projects and scientific events, supporting the next generation of researchers, and enhancing cross-sector collaboration.					
Volkswagen Foundation	Support across borders The Foundation helps to overcome borders – both between academic disciplines and research fields, as well as between countries and cultures.	Yes	Yes	No	https://portal.volkswagenstiftung.de/search/newSearchForm.do	Global health funder list by collegeau

For peer review only

Supplementary file

Supplement S5: Screening studies for PSE reporting manual

Definition of Patient and Stakeholder Engagement (PSE)
Patient and Stakeholder Engagement (PSE) refers to the involvement of patients and other relevant stakeholders (such as caregivers, healthcare providers, patient advocacy groups, policymakers, and the community) in any phase of the clinical study process. This involvement may include contributions to study design, recruitment strategies, data collection, analysis, dissemination of results, or policy integration.

Coding Guidelines
Inclusion Criteria for PSE Reporting
- Any mention of patients or stakeholders being involved in the planning, design, implementation, or dissemination phases of the study.
- Descriptions of how patient or stakeholder input was solicited, used, or valued in the research process.
- References to consultations, focus groups, advisory panels, or committees that include patients or other stakeholders.
- Reports on specific roles or tasks undertaken by patients or stakeholders in the study.
- Mentions of the impact or influence of patient or stakeholder contributions on the study.

Exclusion Criteria
- General mentions of patients or stakeholders without specific reference to their engagement in the research process.
- Solely describing the population characteristics without detailing engagement activities.
- Routine patient consent or standard recruitment processes without additional engagement activities.

Examples of PSE Reporting
Study Design
- "Patients were involved in the development of the study protocol and the selection of outcome measures."
- "A stakeholder advisory panel was consulted to refine the research questions and methodology."

Recruitment
- "Community leaders assisted in developing culturally sensitive recruitment strategies."
- "Patient advocates helped design recruitment materials to ensure they were accessible and understandable."

Data Collection
- "Patients participated in pilot testing the data collection instruments."
- "Stakeholders provided feedback on the data collection process to improve participant experience."

Analysis and Interpretation
- "Patients and caregivers contributed to the interpretation of the study results."
- "Stakeholder input was sought to contextualize the findings and suggest practical implications."

Dissemination
- "Patients co-authored the publication and were involved in presenting the findings at conferences."
- "Stakeholders helped develop dissemination materials targeted at specific communities."

Policy and Practice Integration:

For peer review only

Supplementary file

- "Policy makers were engaged to discuss the potential implications of the study findings for health policy."
- "Stakeholders participated in workshops to translate research findings into practice guidelines."

Coding Procedure:

Read Each Paper Thoroughly: Focus on sections like the introduction, methods, results, discussion, and acknowledgments where PSE might be mentioned.

Highlight Relevant Passages: Use the definition and examples provided to identify and highlight text passages that mention PSE.

Document and Categorize: Record the presence of PSE reporting and categorize the type of engagement (e.g., study design, recruitment, data collection, etc.). Copy-paste PSE mentions in the same excel file.

For peer review only
